# Amino acid removal during hemodialysis can be compensated for by protein ingestion and is not compromised by intradialytic exercise: a randomized controlled crossover trial

**DOI:** 10.1093/ajcn/nqab274

**Published:** 2021-09-12

**Authors:** Floris K Hendriks, Joey S J Smeets, Janneau M X van Kranenburg, Natascha J H Broers, Frank M van der Sande, Lex B Verdijk, Jeroen P Kooman, Luc J C van Loon

**Affiliations:** Department of Human Biology, NUTRIM School of Nutrition and Translational Research in Metabolism, Maastricht University, Maastricht, The Netherlands; Department of Internal Medicine, NUTRIM School of Nutrition and Translational Research in Metabolism, Maastricht University, Maastricht, The Netherlands; Department of Human Biology, NUTRIM School of Nutrition and Translational Research in Metabolism, Maastricht University, Maastricht, The Netherlands; Department of Human Biology, NUTRIM School of Nutrition and Translational Research in Metabolism, Maastricht University, Maastricht, The Netherlands; Department of Internal Medicine, NUTRIM School of Nutrition and Translational Research in Metabolism, Maastricht University, Maastricht, The Netherlands; Division of Nephrology, Department of Internal Medicine, Maastricht University Medical Centre+, Maastricht, The Netherlands; Department of Human Biology, NUTRIM School of Nutrition and Translational Research in Metabolism, Maastricht University, Maastricht, The Netherlands; Department of Internal Medicine, NUTRIM School of Nutrition and Translational Research in Metabolism, Maastricht University, Maastricht, The Netherlands; Division of Nephrology, Department of Internal Medicine, Maastricht University Medical Centre+, Maastricht, The Netherlands; Department of Human Biology, NUTRIM School of Nutrition and Translational Research in Metabolism, Maastricht University, Maastricht, The Netherlands

**Keywords:** hemodialysis, protein, exercise, end-stage renal disease, amino acids, physical activity, supplementation, muscle

## Abstract

**Background:**

Patients with end-stage renal disease (ESRD) undergoing hemodialysis experience a rapid decline in skeletal muscle mass and strength. Hemodialysis removes amino acids (AAs) from the circulation, thereby lowering plasma AA concentrations and stimulating proteolysis.

**Objectives:**

In the present study, we evaluate the impact of intradialytic protein ingestion at rest and following exercise on AA removal and plasma AA availability in patients with ESRD.

**Methods:**

Ten patients (age: 65 ± 16 y, male/female: 8/2, BMI: 24.2 ± 4.8 kg/m^2^, serum albumin: 3.4 ± 0.3 g/dL) with ESRD undergoing hemodialysis participated in this randomized controlled crossover trial. During 4 hemodialysis sessions, patients were assigned to ingest 40 g protein or a placebo 60 min after initiation, both at rest (PRO and PLA, respectively) and following exercise (PRO + EX and PLA + EX, respectively). Spent dialysate and blood samples were collected every 30 min throughout hemodialysis to assess AA removal and plasma AA availability.

**Results:**

Plasma AA concentrations declined by 26.1 ± 4.5% within 30 min after hemodialysis initiation during all interventions (*P* < 0.001, η^2^*_p_* > 0.79). Protein ingestion, but not intradialytic exercise, increased AA removal throughout hemodialysis (9.8 ± 2.0, 10.2 ± 1.6, 16.7 ± 2.2, and 17.3 ± 2.3 g during PLA, PLA + EX, PRO, and PRO + EX interventions, respectively; protein effect *P* < 0.001, η^2^*_p_* = 0.97; exercise effect *P* = 0.32, η^2^*_p_* = 0.11). Protein ingestion increased plasma AA concentrations until the end of hemodialysis, whereas placebo ingestion resulted in decreased plasma AA concentrations (time effect *P* < 0.001, η^2^*_p_* > 0.84). Plasma AA availability (incremental AUC) was greater during PRO and PRO + EX interventions (49 ± 87 and 70 ± 34 mmol/L/240 min, respectively) compared with PLA and PLA + EX interventions (–227 ± 54 and –208 ± 68 mmol/L/240 min, respectively; protein effect *P* < 0.001, η^2^*_p_* = 0.98; exercise effect *P* = 0.21, η^2^*_p_* = 0.16).

**Conclusions:**

Protein ingestion during hemodialysis compensates for AA removal and increases plasma AA availability both at rest and during recovery from intradialytic exercise. Intradialytic exercise does not compromise AA removal or reduce plasma AA availability during hemodialysis in a postabsorptive or postprandial state.

See corresponding editorial on page 1886.

## Introduction

Low muscle mass and strength are frequently observed among patients with end-stage renal disease (ESRD) undergoing hemodialysis, which leads to severe impairments in their physical function (1–4). Hemodialysis itself is considered a key factor responsible for the accelerated loss of muscle mass and strength in patients with ESRD (5, 6). Usually, patients undergo three 4-h hemodialysis sessions per week to remove metabolic waste products and excess fluids from their body. We (7) as well as others (8, 9) have reported that hemodialysis removes a considerable amount of amino acids (AAs) from the circulation, thereby lowering plasma AA concentrations. This decline in plasma AA availability is suggested to stimulate proteolysis, which further contributes to the loss of muscle mass in patients receiving chronic hemodialysis treatment (10, 11).

Recently, we have shown that ∼12 g AAs are removed from the circulation during a single hemodialysis session (7). It has been suggested that provision of protein-rich meals or supplements is warranted to compensate for AA removal during hemodialysis (8, 12, 13). Ingested protein is digested and AAs are absorbed in the gut, with 40–70% of the protein-derived AAs being released into the circulation within the next 3–6 h (14–16). However, a postprandial increase in plasma AA concentrations during hemodialysis leads to a greater plasma–dialysate diffusion gradient and, as such, greater AA removal (7, 8). Due to this greater AA removal, the efficacy of protein ingestion to compensate for plasma AA removal during hemodialysis remains to be determined. We hypothesize that ingestion of 40 g protein during hemodialysis will suffice to compensate for AA removal and, as such, prevent reduced plasma AA availability.

Besides protein ingestion, intradialytic exercise (exercise during hemodialysis) has been proposed as an effective strategy to improve physical function in patients on chronic hemodialysis treatment (17, 18). Intradialytic exercise is usually performed at a low to moderate intensity using a cycle ergometer placed in front of the treatment chair or through group-based physical activity sessions (17, 19, 20). However, the potency of intradialytic exercise to support muscle maintenance is still a matter of debate (21–23). It has been suggested that intradialytic exercise may actually enhance hemodialysis-initiated proteolysis and, as such, could even compromise muscle conditioning (21, 24). We hypothesize that intradialytic exercise leads to greater AA removal during hemodialysis in a postprandial and postabsorptive state.

The present study evaluates the impact of protein ingestion during hemodialysis at rest and during recovery from exercise on AA removal and plasma AA availability in patients with ESRD. Ten patients with ESRD receiving chronic hemodialysis treatment were selected to participate in a randomized crossover design. This study provides a complete insight into the impact of both protein ingestion and intradialytic exercise on AA removal and plasma AA availability throughout hemodialysis in patients with ESRD.

### Patients

Ten patients with ESRD and well-functioning arteriovenous shunts, undergoing hemodialysis in the morning or afternoon for at least 3 mo, were recruited between March 2019 and August 2020 through the outpatient population visiting the dialysis department of Maastricht University Medical Centre+, Maastricht, The Netherlands [see **Supplementary File 1**(**Supplementary Figure 1** for the Consolidated Standards of Reporting Trials flow diagram)]. Patients with an active infection, cognitive disorder, intolerance to food ingestion during hemodialysis, contraindication to intradialytic exercise, or missed hemodialysis session in the past month prior to the study period were excluded. After patients expressed willingness to participate to their nephrologist, they were informed by an investigator about the purpose of the study, experimental procedures, and possible risks prior to signing written informed consent. The Medical Research Ethics Committee Academic Hospital Maastricht/Maastricht University (NL65880.068.18) and the Hospital Board of the Academic Hospital Maastricht approved the current study, and it was registered prospectively at the Netherlands Trial Register (NL7152). The present study design complies with the ethical standards stated in the latest version of the Declaration of Helsinki of 1975 as revised in October 2013.

## Methods

### Pretesting

A pretesting session was scheduled during routine hemodialysis at least 1 wk before the first test day to familiarize patients with intradialytic exercise and determine exercise capacity. In addition, patients’ medical history, physical examinations, laboratory analysis results, and hemodialysis regimen were registered. A dialysis cycle ergometer (Thera Riser; Medica Medizintechnik GmbH) was placed in front of the treatment chair and adjusted until the patient was positioned properly. Blood pressure, heart rate, and an electrocardiogram were recorded and directly assessed for abnormalities by a physician throughout intradialytic exercise performance. After a 5-min warmup, the resistance level of the dialysis cycle ergometer was increased until patients reported a score between 12 and 15 on the 6- to 20-point Borg Ratings of Perceived Exertion scale (25). Subsequently, patients were instructed to continue cycling at the same resistance level for 10 min. When patients reported a score <12 or >15 on the 6- to 20-point Borg Ratings of Perceived Exertion scale, the resistance level was adjusted accordingly. The resistance level at which patients succeeded to perform 10 min of moderate-intensity exercise was used for the exercise protocol during test days.

### Dietary intake and physical activity

All patients refrained from any sort of strenuous physical activity 48 h prior to each test day. Patients who underwent hemodialysis in the morning reported in an overnight fasted state. Those who underwent hemodialysis in the afternoon consumed the same standardized breakfast at least 3 h before initiation of their hemodialysis session (providing ∼250 kcal, with carbohydrate, fat, and protein providing 65, 23, and 12% of its energy content, respectively). Thereafter, patients were instructed to remain fasted and avoid caffeine consumption until the end of the experimental protocol but were allowed to ingest water ad libitum. During each test day, dietary intake records were acquired through a 24-h food recall questionnaire. Furthermore, patients filled out a food diary and wore a SenseWear Pro 3 armband (Bodymedia) for 6 d between the first and second test days to assess habitual dietary intake and physical activity levels. A licensed dietitian carefully instructed patients on how to perform the 24-h food recall questionnaires and 6-d food diary. All ingested foods and beverages were reported in household measurements or specified as portion sizes. Subsequently, energy and macronutrient intake were calculated using free available software from the Dutch Nutrition Centre (http://mijn.voedingscentrum.nl) based on product specifications provided by food suppliers and the Dutch Food Consumption Database 2019 (26).

### Study design

**FIGURE 1:**
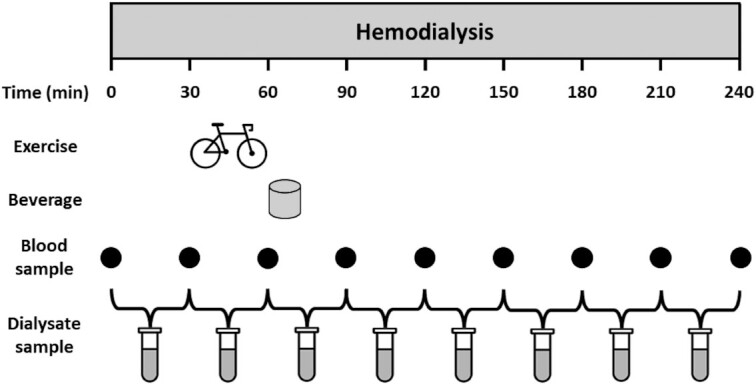
Schematic representation of study protocol. *t* = 0 min represents the start of the hemodialysis session. During 4 hemodialysis sessions, patients ingested 40 g protein or placebo both at rest and during recovery from intradialytic exercise in a randomized crossover design. This figure represents test days for the PLA + EX and PRO + EX interventions. The study protocol for PLA and PRO interventions was similar but without intradialytic exercise. PLA, placebo; PLA + EX, placebo and exercise; PRO, protein; PRO + EX, protein and exercise.

During 4 hemodialysis sessions, separated by a washout period of at least 1 wk, all patients were assigned to ingest a placebo (PLA) or protein (PRO) beverage both in a rested state as well as following 30 min of intradialytic exercise (PLA + EX and PRO + EX, respectively) in a randomized crossover design. The crossover design was chosen to minimize variability of outcome parameters in this heterogeneous population. An overview of test days, which were scheduled during patients’ second or third weekly hemodialysis session, is provided in **Figure 1**. Patients were randomly assigned to an order of interventions by an independent researcher using an online randomizer (http://www.randomizer.org), and the randomization order of test beverages was not shared with investigators, study staff, or participants until all procedures and statistical analyses of the primary and secondary outcomes were complete. The independent researcher was responsible for the preparation of test beverages, which were numbered according to participant and test day number before handing them to an investigator. The protein beverage contained 40 g milk protein concentrate (Refit MPC 80; Friesland Campina) and 2 non-aspartame-containing sweeteners (Natrena; Douwe Egberts) dissolved in 300 mL water. The placebo beverage contained only the 2 sweeteners dissolved in 300 mL water. The independent researcher shared the order of exercise performance during test days with the investigators after pretesting was completed. Although patients were blinded to the order of exercise performance, it was not possible to conceal the intervention during test days due to the nature of the exercise intervention. Patients started the intradialytic exercise by performing a 5-min warmup on the dialysis cycle ergometer, during which they were instructed not to surpass a score of 9 on the 6- to 20-point Borg Ratings of Perceived Exertion scale. Subsequently, the resistance level was increased to the previously determined value, and patients continued cycling for 20 min. At the end of the intradialytic exercise, patients performed a cool-down consisting of 3 min of cycling with a score between 9 and 12 and the last 2 min with a score below 9 on the 6- to 20-point Borg Ratings of Perceived Exertion scale.

### Hemodialysis treatment

Patients’ prescribed blood (300–400 mL/min) and dialysate flow rates (500–600 mL/min), dialysate composition, dialysis modality, and dialysis membranes were used during hemodialysis and kept constant throughout all test days. Desired ultrafiltration volume was determined by the treating nephrologist for each hemodialysis session. Patients were dialyzed through a well-functioning arteriovenous shunt in the arm using polysulfone (*n* = 4; FX-100; Fresenius Medical Care), polynephron (*n* = 3; Elisio 17H; Nipro Medical Corporation), and triacetate (*n* = 2; SUREFLUX 19 L and *n* = 1; SURFLUX 19UX; Nipro Medical Corporation) membranes.

### Experimental protocol

After patients arrived at the dialysis department, their weight was recorded and a Body Composition Monitor (Fresenius Medical Care) was used to assess their body composition, as described before (27). Subsequently, the arteriovenous shunt was checked for recirculation and used to collect arterial plasma samples for AA concentrations analyses. After initiation of hemodialysis (*t* = 0 min), plasma samples were collected from the arterial line with 30-min intervals (at *t* = 30, 60, 90, 120, 150, 180, and 210 min) and spent dialysate was collected continuously in a container at a rate of 1.0 L/h using a reversed injection pump (Alaris GW). Every 30 min, these containers were replaced (at *t* = 30, 60, 90, 120, 150, 180, 210, and 240 min) and a homogenized sample of the spent dialysate collected over each 30-min period was obtained. Blood pressure and heart rate were measured frequently throughout hemodialysis. During the sessions including intradialytic exercise, patients started cycling 30 min after hemodialysis initiation (*t* = 30 min), and additional measurements of blood pressure and heart rate were performed during and after exercise (at *t* = 40, 50, and 70 min). In all sessions, patients ingested the test beverage 1 h after hemodialysis initiation (*t* = 60 min) and remained in a rested state thereafter. Directly after hemodialysis (*t* = 240 min), a plasma sample was collected from the arterial side of the arteriovenous shunt. Following the experimental procedures, patients consumed a standard meal before leaving the dialysis department.

### Plasma amino acid analysis

Plasma samples were collected in EDTA-containing tubes and centrifuged at 3500 × *g* at 4°C for 10 min to obtain plasma. Aliquots of plasma were frozen in liquid nitrogen and stored in a freezer at –80°C until further analysis. For determination of plasma AA concentrations, 50 µL blood plasma was deproteinized using 100 µL 10% 5-sulfosalicylic acid with 50 µM of the metabolomics AA mix MSK-A2 internal standard (Cambridge Isotope Laboratories). Subsequently, 50 µL ultra-pure demineralized water was added and the samples were centrifuged. Thereafter, 10 µL supernatant was added to 70 µL Borate reaction buffer (Waters). In addition, 20 µL AccQ-Tag derivatizing reagent solution (Waters) was added, and the mixture was subsequently heated to 55°C for 10 min. AA profiles in the derivative were determined by ultra-performance liquid chromatography mass spectrometry (UPLC-MS; ACQUITY UPLC H-Class with QDa; Waters) as described previously (28).

### Dialysate amino acid analysis

Spent dialysate samples were collected in sterile tubes, immediately frozen in liquid nitrogen, and stored in a freezer at –80°C until further analysis. These samples were concentrated through freeze-drying 25 mL of the sample and dissolving the dried product in 5.0 mL 0.1 M hydrogen chloride. After homogenization, the concentrated samples were processed in the same manner as plasma samples, and AA profiles were determined through UPLC-MS.

### Statistical analysis

All data are expressed as means ± SDs unless indicated otherwise. A power calculation was performed with differences in incremental AUC (iAUC) of plasma AA concentrations as the primary outcome measure. A sample size of 10 participants, including a 20% dropout rate, was calculated using a power of 80%, a significance level of 0.025 to compensate for the crossover design with 2 interventions, and a difference in iAUCs of 13% between treatments with a standard deviation of 11% based on a previous study from our laboratory (29). Secondary outcome parameters include plasma and spent dialysate total amino acid (TAA), branched-chain amino acid (BCAA), nonessential amino acid (NEAA), and essential amino acid (EAA) concentrations, AA removal, correlations between AA concentrations in plasma and spent dialysate, habitual dietary energy and macronutrient intake, and habitual physical activity levels. After the randomization order of test beverages was shared with investigators, hemodialysis parameters and pre-hemodialysis weight were compared between interventions to identify possible confounders. Normal distribution of all parameters was verified by Shapiro–Wilk tests (*P* > 0.05). No major violations for specific 3-factor repeated-measures ANOVA assumptions were observed, and in case of nonsphericity, the Greenhouse–Geisser correction was used. Potential differences in AA concentrations over time were assessed using 3-factor repeated-measures ANOVA with time, protein ingestion (yes/no), and exercise (yes/no) as within-subject factors. AA removal, the iAUC of plasma AA concentrations representing the *t* = 0–240 min period, hemodialysis parameters, and pre-hemodialysis weight were analyzed by 2-factor repeated-measures ANOVA with protein ingestion (yes/no) and exercise (yes/no) as within-subject variables. If a statistically significant interaction was found, 2-factor ANOVAs and/or subsequent paired-samples *t* tests were performed. In case of significant time effects, Bonferroni post hoc analyses were performed to locate the effects. Dietary energy and macronutrient intake and physical activity values on dialysis days and nondialysis days were compared using paired-samples *t* tests. Correlations between AA concentrations in spent dialysate and the average of the 2 corresponding plasma samples (e.g., *t* = 30 and *t* = 60 min for spent dialysate collected between *t* = 30 and 60 min) were assessed through determining Pearson correlation coefficients. Effect sizes were calculated for plasma and spent dialysate AA concentrations using partial η squared (η^2^*_p_*) for ANOVA comparisons. Statistical significance was set at *P* < 0.05. All analyses were performed using SPSS statistics software (version 24.0; IBM Corp.).

## Results

### Patients’ characteristics

All 10 included patients with ESRD completed 4 test days. Patients’ baseline characteristics are presented in **Table 1**. Six patients were anuric, 1 patient was oliguric, and 3 patients had a remaining diuresis >400 mL/24 h. No differences were observed between PLA, PLA + EX, PRO, and PRO + EX interventions in ultrafiltration volume (1.24 ± 1.01, 1.47 ± 1.27, 1.23 ± 1.08, and 1.41 ± 1.24 L, respectively; *P* > 0.05), dialysis adequacy (equilibrated Kt/V: 1.45 ± 0.22, 1.53 ± 0.22, 1.57 ± 0.27, and 1.48 ± 0.22, respectively; *P* > 0.05), and pre-hemodialysis weight (71.9 ± 14.3, 72.6 ± 14.0, 72.2 ± 13.9, and 71.9 ± 14.1 kg, respectively; *P* > 0.05).

**TABLE 1:** Patients’ characteristics^1^

Characteristic	Value
Age, y	65 ± 16
Sex, male/female	8/2
Cause of end-stage renal disease	
Glomerular	5
Vascular	4
Unknown	1
Dialysis vintage, mo	36 ± 23
Dialysis timing, morning/afternoon	5/5
Height, m	1.72 ± 0.13
Weight, kg	71.0 ± 13.6
BMI, kg/m^2^	24.2 ± 4.8
Lean tissue index, kg/m^2^	13.3 ± 2.5
Fat tissue index, kg/m^2^	10.4 ± 5.9
Serum albumin, g/dL	3.4 ± 0.3
C-reactive protein, mg/L	7 ± 6

1 Continuous and categorical values are expressed as means ± SDs and counts, respectively, *n* = 10.

### Habitual dietary intake and physical activity

Two patients declined to fill out a food diary, and 2 patients did not wear the SenseWear armband correctly. Reported habitual dietary energy and protein intakes averaged 25.9 ± 6.0 kcal/kg body weight/d and 1.0 ± 0.3 g/kg body weight/d, respectively. No statistical differences were observed in habitual energy and macronutrient intake between nondialysis and dialysis days (**Table 2**). In contrast, activity-related energy expenditure was lower on dialysis days (7 ± 9 kcal/kg body weight) compared with nondialysis days (12 ± 13 kcal/kg body weight; *P* = 0.04). However, the differences between physical activity duration and number of steps taken on nondialysis and dialysis days were not statistically significant (Table 2).

**TABLE 2:** Habitual food intake and physical activity on dialysis and nondialysis days^1^

Characteristic	Daily mean	DD	Non-DD	*P*
Habitual intake				
Energy, kcal	1874 ± 605	2074 ± 812	1763 ± 433	0.29
Energy, kcal/kg body weight	25.9 ± 6.0	28.1 ± 9.5	24.8 ± 6.3	0.24
Carbohydrate, g	217 ± 62	240 ± 83	205 ± 43	0.26
Carbohydrate, g/kg body weight	3.0 ± 0.6	3.3 ± 1.0	2.7 ± 0.4	0.32
Protein, g	73 ± 29	80 ± 37	69 ± 24	0.33
Protein, g/kg body weight	1.0 ± 0.3	1.1 ± 0.4	0.9 ± 0.3	0.19
Fat, g	71 ± 34	81 ± 40	66 ± 30	0.28
Fat, g/kg body weight	1.0 ± 0.3	1.1 ± 0.5	0.9 ± 0.3	0.10
Physical activity				
Number of steps	4202 ± 3943	3575 ± 4739	4515 ± 3535	0.29
Activity-related energy expenditure, kcal/kg	10 ± 12	7 ± 9	12 ± 13	0.04
Moderate-vigorous activity duration, min	145 ± 162	102 ± 127	166 ± 174	0.12

1 All values are expressed as means ± SDs, *n* = 8. Data of dialysis days and nondialysis days were compared using paired-samples *t* tests. Daily mean values represent the average of dialysis and nondialysis days, measured over a 6-d period. DD, dialysis day.

### Plasma amino acid concentrations

**FIGURE 2:**
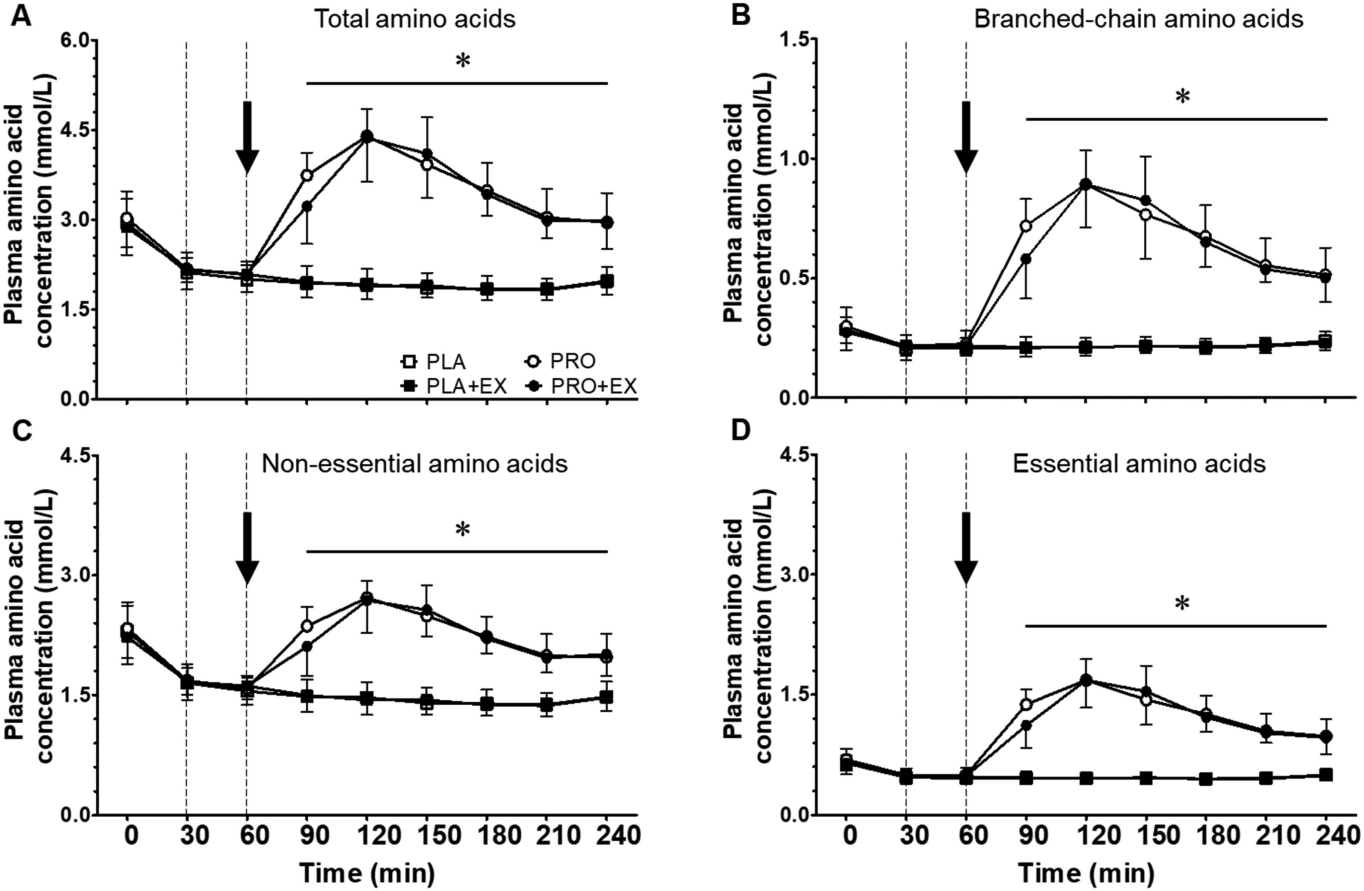
Plasma (A) total, (B) branched-chain, (C) nonessential, and (D) essential amino acid concentrations throughout hemodialysis at rest and following exercise with and without protein ingestion. The dotted lines represent the start and end of intradialytic exercise, and the arrow represents the ingestion of the test beverage. Values are expressed as means ± SDs, *n* = 10 for all values. Data were analyzed with a 3-factor repeated-measures ANOVA with time, protein ingestion (yes/no), and exercise (yes/no) as within-subject variables, and separate analysis was performed when a significant interaction was detected. Time × protein interaction *P* < 0.05. *Protein interventions significantly different from placebo interventions (protein effect *P* ≤ 0.001). PLA, placebo; PLA + EX, placebo and exercise; PRO, protein; PRO + EX, protein and exercise.

**FIGURE 3:**
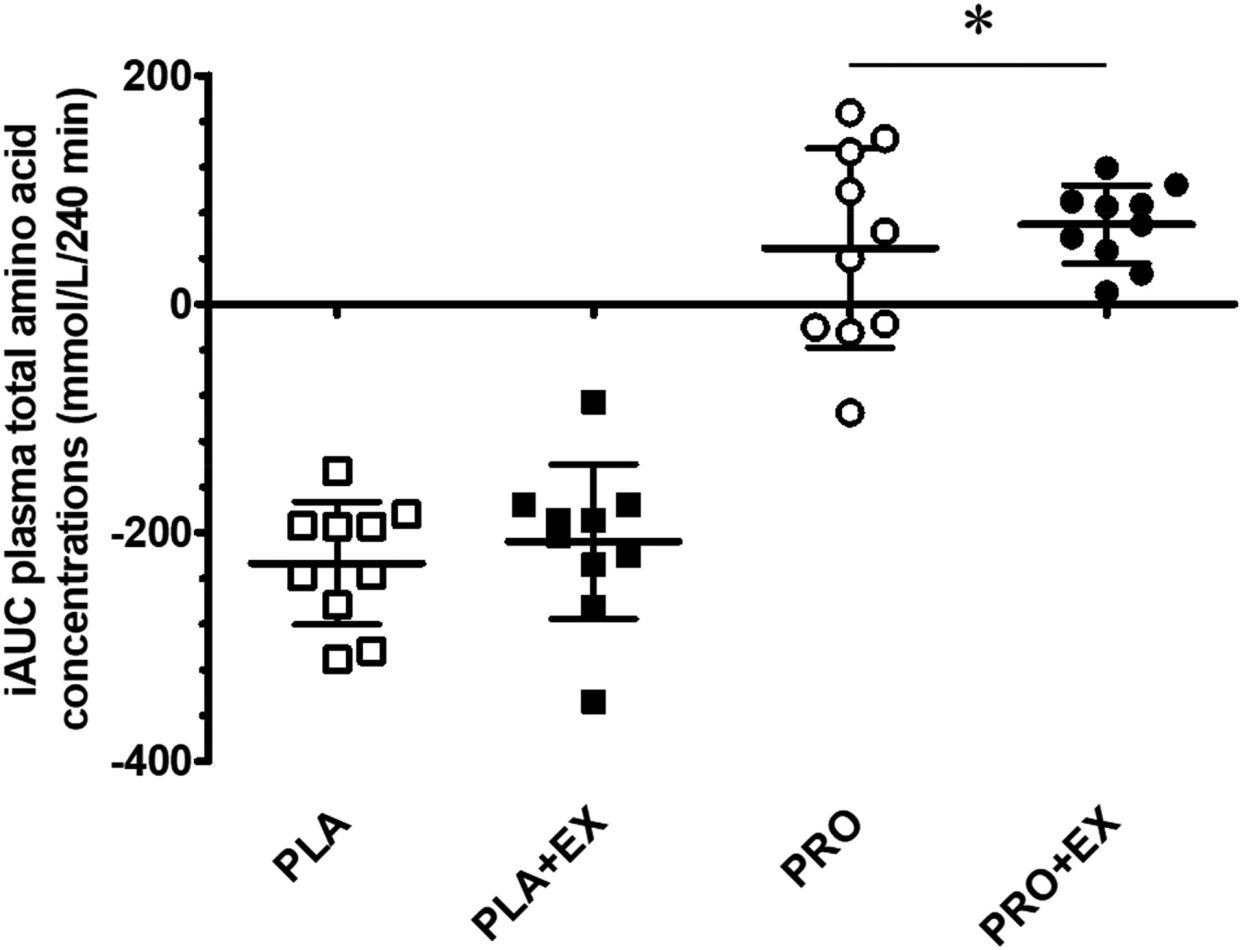
Incremental AUC of plasma total amino acid concentrations throughout hemodialysis at rest and following exercise with and without protein ingestion. The incremental AUC was calculated over the 240-min hemodialysis period. Squares and circles represent individual data points, and bars represent group means ± SDs, *n* = 10. Data were analyzed with a 2-factor repeated-measures ANOVA with protein ingestion (yes/no) and exercise (yes/no) as within-subject variables. *Significantly different from placebo interventions (protein effect *P* < 0.001). iAUC, incremental AUC; PLA, placebo; PLA + EX, placebo and exercise; PRO, protein; PRO + EX, protein and exercise.

Pre-hemodialysis plasma TAA concentrations averaged 2.93 ± 0.40 mmol/L, with no differences between interventions (**Figure 2**; *P* > 0.05). A significant time × protein interaction was observed for plasma TAA concentrations throughout hemodialysis (*P* < 0.001, η^2^*_p_* = 0.87). Separate analyses showed that following hemodialysis initiation, plasma TAA concentrations decreased substantially during the first 30 min (*P* < 0.001, η^2^*_p_* > 0.79 for all interventions). During PLA and PLA + EX interventions, plasma TAA concentrations continued to decrease over time to 1.84 ± 0.18 and 1.83 ± 0.16 mmol/L at *t* = 210 min, respectively (time effect *P* < 0.001, η^2^*_p_* = 0.69). Plasma TAA concentrations increased following protein ingestion during PRO and PRO + EX interventions (time effect *P* < 0.001, η^2^*_p_* = 0.80). Peak plasma TAA concentrations were observed 60 min after protein ingestion (*t* = 120 min), with no differences between PRO and PRO + EX interventions (4.40 ± 0.45 and 4.37 ± 0.73 mmol/L, respectively; protein × exercise interaction *P* = 0.34, η^2^*_p_* = 0.10). In line with these data, an effect of protein ingestion (protein effect *P* < 0.001, η^2^*_p_* = 0.98) but no effect of intradialytic exercise (exercise effect *P* = 0.21, η^2^*_p_* = 0.16) was observed on the iAUC of plasma TAA concentrations during PLA, PLA + EX, PRO, and PRO + EX interventions (**Figure 3**; –227 ± 54, –208 ± 68, 49 ± 87, and 70 ± 34 mmol/L/240 min, respectively). As shown in Figure 2, plasma BCAA, NEAA, and EAA concentrations throughout hemodialysis responded in the same manner as plasma TAA concentrations to protein ingestion and intradialytic exercise. Plasma concentrations and iAUCs of individual AAs throughout hemodialysis are provided in **Supplementary File 2**.

### Spent dialysate amino acid concentrations

**FIGURE 4:**
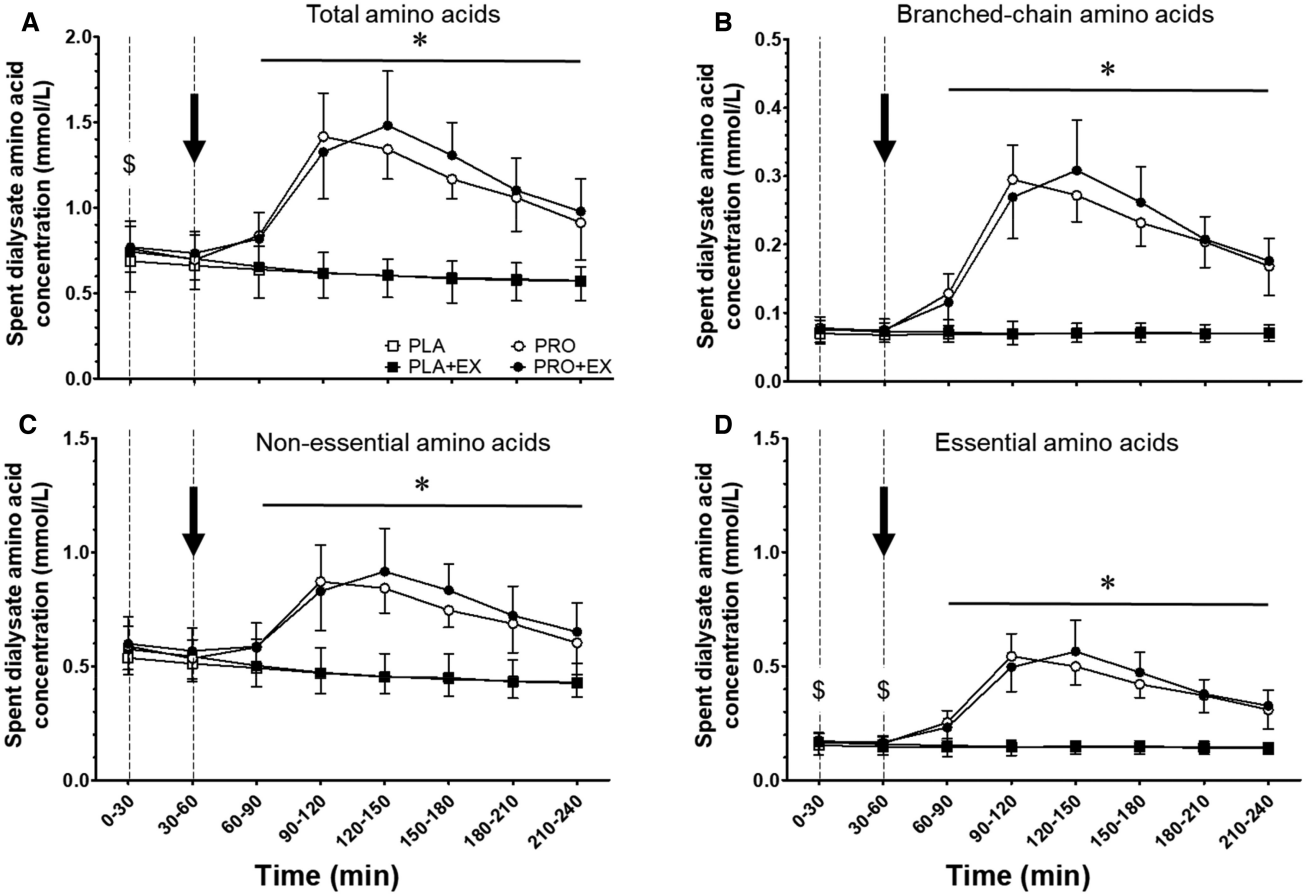
Spent dialysate (A) total, (B) branched-chain, (C) nonessential, and (D) essential amino acid concentrations throughout hemodialysis at rest and following exercise with and without protein ingestion. The dotted lines represent the start and end of intradialytic exercise, and the arrow represents the ingestion of the test beverage. Values are expressed as means ± SDs, *n* = 10 for all values. Data were analyzed with a 3-factor repeated-measures ANOVA with time, protein ingestion (yes/no), and exercise (yes/no) as within-subject variables, and separate analysis was performed when a significant interaction was detected. Time × protein interaction *P* < 0.05. ^$^Protein interventions significantly different from placebo interventions (protein effect *P* < 0.05). *Protein interventions significantly different from placebo interventions (protein effect *P* < 0.001). PLA, placebo; PLA + EX, placebo and exercise; PRO, protein; PRO + EX, protein and exercise.

**FIGURE 5:**
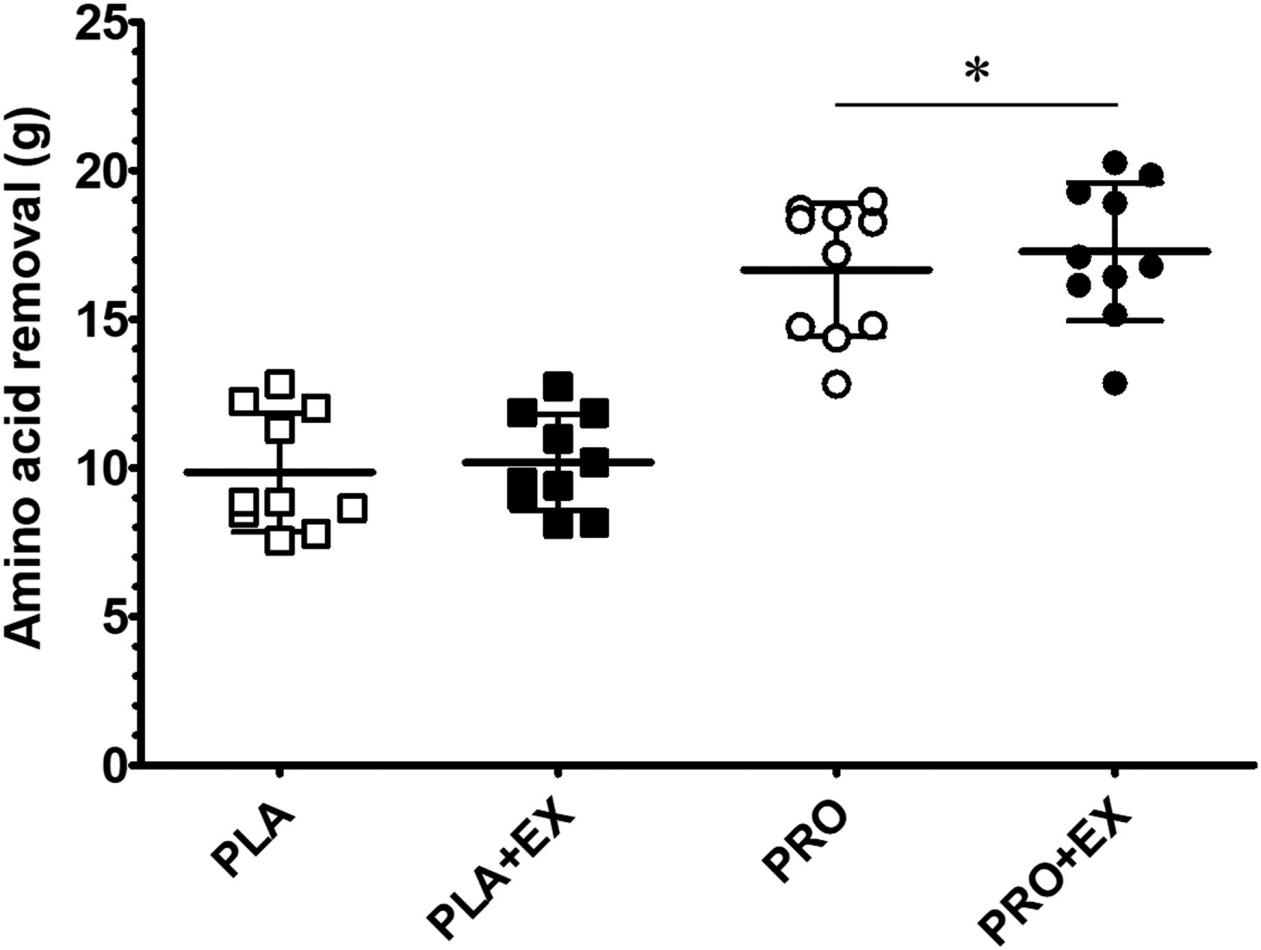
Total amino acid removal throughout hemodialysis at rest and following exercise with and without protein ingestion. Squares and circles represent individual data points, and bars represent group means ± SDs, *n* = 10. Data were analyzed with a 2-factor repeated-measures ANOVA with protein ingestion (yes/no) and exercise (yes/no) as within-subject variables. *Significantly different from placebo interventions (protein effect *P* < 0.001). PLA, placebo; PLA + EX, placebo and exercise; PRO, protein; PRO + EX, protein and exercise.

AA concentrations in the spent dialysate are presented in **Figure 4**. Spent dialysate AA concentrations correlated well with circulating plasma AA concentrations (Pearson *r* = 0.91, *P* < 0.001). A significant time × protein interaction was observed for spent dialysate TAA concentrations throughout hemodialysis (*P* < 0.001, η^2^*_p_* = 0.89). Spent dialysate TAA concentrations decreased over time during PLA and PLA + EX interventions toward 0.57 ± 0.11 and 0.57 ± 0.08 mmol/L during the last 30-min period of hemodialysis, respectively (*P* = 0.005, η^2^*_p_* = 0.77). In contrast, spent dialysate TAA concentrations significantly increased following protein ingestion during PRO and PRO + EX interventions and remained elevated until the end of hemodialysis (time effect *P* < 0.05, η^2^*_p_* = 0.87). Protein ingestion significantly increased AA removal during PRO and PRO + EX compared with PLA and PLA + EX interventions (**Figure 5**; 16.7 ± 2.2 and 17.3 ± 2.3 compared with 9.8 ± 2.0 and 10.2 ± 1.6 g, respectively; protein effect *P* < 0.001, η^2^*_p_* = 0.97). Intradialytic exercise did not modulate AA removal (exercise effect *P* = 0.32, η^2^*_p_* = 0.11). Furthermore, spent dialysate BCAA, NEAA, and EAA concentrations showed similar perturbations throughout hemodialysis as spent dialysate TAA concentrations (Figure 4). Spent dialysate concentrations and removal of individual AAs throughout hemodialysis are provided in Supplementary File 2.

## Discussion

In this randomized controlled crossover study, we observed that AAs are removed from the circulation during hemodialysis, thereby lowering plasma AA concentrations in patients with ESRD. Protein ingestion during hemodialysis compensated for AA removal and prevented a decline in plasma AA availability at rest and during recovery from intradialytic exercise. Exercise performed during hemodialysis did not modulate AA removal or plasma AA availability in patients with ESRD.

Hemodialysis treatment is essential for patients with ESRD as it prevents accumulation of metabolic waste products up to lethal concentrations. However, hemodialysis also removes AAs from the circulation because they, just like metabolic waste products, diffuse through the dialysis membrane (9). In the current study, we observed a substantial decline in circulating plasma TAA concentrations from 2.93 ± 0.40 to 2.16 ± 0.26 mmol/L within 30 min following the initiation of hemodialysis (Figure 2). Such a decrease in plasma AA concentrations has been shown to stimulate proteolysis in peripheral tissues (10, 30–32). Furthermore, by also measuring AA concentrations in the spent dialysate, we were able to assess AA removal throughout the hemodialysis session, which ranged between 7 and 12 g during placebo interventions (Figure 5). This loss is representative of the amount of AAs being released in the circulation following ingestion of a normal meal providing ∼20 g protein (15). As a consequence, AA removal during hemodialysis has been proposed to represent a key factor responsible for the accelerated loss of muscle mass in patients with ESRD (6, 33, 34).

We first assessed the impact of protein ingestion during hemodialysis as a means to compensate for AA removal and, as such, to support muscle maintenance. To overcome reduced protein digestion and absorption kinetics of patients receiving chronic hemodialysis treatment as well as increased AA removal following protein ingestion during hemodialysis (7, 15), we provided all patients with a bolus of 40 g protein. Ingestion of 40 g protein during hemodialysis elevated plasma AA concentrations (Figure 2). This stimulated AA removal, resulting in ∼8 g more AAs being removed from the circulation compared with placebo ingestion. Despite the greater AA removal (Figure 5), plasma AA availability was strongly elevated following protein ingestion (Figure 3). Preventing a decline in plasma AA availability throughout hemodialysis has been reported to attenuate muscle proteolysis during and after hemodialysis (8, 11, 30). We conclude that ingestion of 40 g protein is sufficient to compensate for intradialytic AA removal, prevent a decline in plasma AA concentrations, and increase plasma AA availability. Especially the latter may be of key importance to achieve a positive muscle net protein balance during hemodialysis.

Another key strategy to support muscle maintenance in patients on chronic hemodialysis treatment is the implementation of physical activity or exercise interventions (35, 36). Previous work has shown various benefits of lifestyle intervention in patients with chronic kidney disease, including those undergoing hemodialysis (37, 38). However, the effectiveness of these lifestyle intervention programs for patients receiving chronic hemodialysis treatment is typically compromised by low adherence and compliance (39). Exercise intolerance, fatigue, and lack of exercise knowledge often prevent these patients from increasing their physical activity levels (21, 40). Consequently, effective physical activity intervention programs need to be individualized and performed under strict supervision. Therefore, implementation of physical activity or exercise during hemodialysis has been proposed as a practical and efficient intervention strategy as it would be more time efficient for patients and relatively easy to supervise by (para)medical staff (41). Benefits of structured intradialytic exercise performance entail improved aerobic capacity, physical function, health-related quality of life, and better clearance of metabolic waste products during hemodialysis (17, 19, 42, 43). However, it has been suggested that intradialytic exercise without concurrent protein ingestion may actually exacerbate muscle catabolism (44), which could result in even greater AA removal. Therefore, in the present study, we assessed the impact of intradialytic exercise on AA removal and plasma AA availability in both the presence and the absence of protein ingestion. Here, we observed no differences in AA removal during a hemodialysis session with (10.2 ± 1.6 g) or without (9.8 ± 2.0 g) intradialytic exercise (Figure 5). Furthermore, we observed no differences in plasma AA availability due to intradialytic exercise (Figure 3). This implies that intradialytic exercise performed in a postabsorptive state does not necessarily impair the net protein balance during hemodialysis. However, the muscle net protein balance will not become positive when exercise is performed without concomitant protein ingestion (45).

To facilitate the skeletal muscle-adaptive response to exercise, ample availability of circulating AAs is required (46, 47). Therefore, intradialytic exercise combined with protein ingestion to compensate for AA removal and increase plasma AA availability represents a preferred strategy. So far, there have not been any studies to assess the impact of intradialytic exercise and protein ingestion on AA removal and plasma AA availability. In line with our findings described above, we observed that ingestion of 40 g protein directly after intradialytic exercise increases plasma AA concentrations with levels remaining elevated until the end of the hemodialysis session (Figure 2). As a result, intradialytic exercise did not have any impact on plasma AA availability throughout the 4-h hemodialysis session (Figure 3). Furthermore, intradialytic exercise did not significantly increase AA removal following protein ingestion (16.6 ± 2.2 compared with 17.3 ± 2.3 g in PRO and PRO + EX, respectively; Figure 5). Therefore, protein ingestion increases plasma AA availability during hemodialysis, which may create a setting in which hemodialysis-initiated proteolysis is inhibited and muscle conditioning after exercise performance is supported.

Combining protein ingestion and exercise during hemodialysis provides a practical interventional strategy that may help to preserve muscle mass and maintain functional capacity in patients receiving chronic hemodialysis treatment. However, the design of the present study has some limitations. The included patients were volunteers, which may introduce some confounding as the less clinically compromised patients may be more likely to partake. Nonetheless, it is generally hypothesized that malnourished patients undergoing hemodialysis benefit to a greater extent from intradialytic protein supplementation with or without exercise compared with well-nourished patients (44, 48). As we performed statistical analyses of multiple secondary outcomes in the present study, there is an increased risk of a type I error among the secondary outcome parameters. In addition, we assessed the impact of protein ingestion and exercise on plasma AA concentrations and AA removal during hemodialysis sessions, which may or may not necessarily translate to increases in muscle mass or improvements in physical function over a more prolonged treatment period.

So far, long-term intervention studies investigating the effects of intradialytic oral nutritional supplementation with or without exercise training on muscle mass and function have reported equivocal results (24, 44, 49). This may be largely due to exercise intolerance and the low adherence of these patients to lifestyle intervention (39, 40, 50). Furthermore, the uremic and inflammatory milieu in these patients may compromise the capacity of skeletal muscle tissue to properly respond to protein ingestion and exercise training. For example, Jeong et al. (44) reported no improvements in physical function or body composition following 12 months of intradialytic protein ingestion and exercise. More work will be needed to establish the various exercise modalities and adjuvant nutritional support that will effectively support muscle mass maintenance in this heterogeneous population.

In conclusion, protein ingestion during hemodialysis compensates for AA removal and increases plasma AA availability at rest and during recovery from intradialytic exercise. Intradialytic exercise should be combined with protein ingestion to compensate for AA removal during hemodialysis and, as such, allow a setting that may support muscle reconditioning in patients with ESRD.

## Supplementary Material

nqab274_Supplemental_File

## Data Availability

Data described in the manuscript, code book, and analytic code will be made available upon request pending application and approval.

## Notes

Supported by a grant from the NUTRIM NWO Graduate Programme.

Supplementary File 1 (Supplementary Figure 1) and Supplementary File 2 (Supplementary Figures 2–21) are available from the “Supplementary data” link in the online posting of the article and from the same link in the online table of contents at https://academic.oup.com/ajcn/.

Address correspondence to LJCL (e-mail: L.vanLoon@maastrichtuniversity.nl).

Abbreviations used: AA, amino acid; BCAA, branched-chain amino acid; ESRD, end-stage renal disease; EAA, essential amino acid; iAUC, incremental AUC; NEAA, nonessential amino acid; PLA, placebo; PLA + EX, placebo and exercise; PRO, protein; PRO + EX, protein and exercise; TAA, total amino acid; UPLC-MS, ultra-performance liquid chromatography mass spectrometry.

## References

[1] Carrero JJ , Thomas F, Nagy K, Arogundade F, Avesani CM, Chan M, Chmielewski M, Cordeiro AC, Espinosa-Cuevas A, Fiaccadori E et al. Global prevalence of protein-energy wasting in kidney disease: a meta-analysis of contemporary observational studies from the International Society of Renal Nutrition and Metabolism. J Ren Nutr. 2018;28(6):380–92. 30348259 10.1053/j.jrn.2018.08.006

[2] Isoyama N , Qureshi AR, Avesani CM, Lindholm B, Barany P, Heimburger O, Cederholm T, Stenvinkel P, Carrero JJ. Comparative associations of muscle mass and muscle strength with mortality in dialysis patients. Clin J Am Soc Nephrol. 2014;9(10):1720–8. 25074839 10.2215/CJN.10261013 PMC4186520

[3] Broers NJ , Usvyat LA, Kooman JP, van der Sande FM, Lacson E Jr, Kotanko P, Maddux FW. Quality of life in dialysis patients: a retrospective cohort study. Nephron. 2015;130(2):105–12. 26044799 10.1159/000430814

[4] Bataille S , Serveaux M, Carreno E, Pedinielli N, Darmon P, Robert A. The diagnosis of sarcopenia is mainly driven by muscle mass in hemodialysis patients. Clin Nutr. 2017;36(6):1654–60. 27816311 10.1016/j.clnu.2016.10.016

[5] Carrero JJ , Stenvinkel P, Cuppari L, Ikizler TA, Kalantar-Zadeh K, Kaysen G, Mitch WE, Price SR, Wanner C, Wang AY et al. Etiology of the protein-energy wasting syndrome in chronic kidney disease: a consensus statement from the International Society of Renal Nutrition and Metabolism (ISRNM). J Ren Nutr. 2013;23(2):77–90. 23428357 10.1053/j.jrn.2013.01.001

[6] Marcelli D , Brand K, Ponce P, Milkowski A, Marelli C, Ok E, Merello Godino JI, Gurevich K, Jirka T, Rosenberger J et al. Longitudinal changes in body composition in patients after initiation of hemodialysis therapy: results from an international cohort. J Ren Nutr. 2016;26(2):72–80. 26627050 10.1053/j.jrn.2015.10.001

[7] Hendriks FK , Smeets JSJ, Broers NJH, van Kranenburg JMX, van der Sande FM, Kooman JP, van Loon LJC. End-stage renal disease patients lose a substantial amount of amino acids during hemodialysis. J Nutr. 2020;150(5):1160–6. 32006029 10.1093/jn/nxaa010 PMC7198312

[8] Veeneman JM , Kingma HA, Boer TS, Stellaard F, De Jong PE, Reijngoud DJ, Huisman RM. Protein intake during hemodialysis maintains a positive whole body protein balance in chronic hemodialysis patients. Am J Physiol Endocrinol Metab. 2003;284(5):E954–65. 12540372 10.1152/ajpendo.00264.2002

[9] Ikizler TA , Flakoll PJ, Parker RA, Hakim RM. Amino acid and albumin losses during hemodialysis. Kidney Int. 1994;46(3):830–7. 7996804 10.1038/ki.1994.339

[10] Ikizler TA , Pupim LB, Brouillette JR, Levenhagen DK, Farmer K, Hakim RM, Flakoll PJ. Hemodialysis stimulates muscle and whole body protein loss and alters substrate oxidation. Am J Physiol Endocrinol Metab. 2002;282(1):E107–16. 11739090 10.1152/ajpendo.2002.282.1.E107

[11] Sundell MB , Cavanaugh KL, Wu P, Shintani A, Hakim RM, Ikizler TA. Oral protein supplementation alone improves anabolism in a dose-dependent manner in chronic hemodialysis patients. J Ren Nutr. 2009;19(5):412–21. 19500999 10.1053/j.jrn.2009.01.019 PMC2758490

[12] Kistler BM , Benner D, Burrowes JD, Campbell KL, Fouque D, Garibotto G, Kopple JD, Kovesdy CP, Rhee CM, Steiber A et al. Eating during hemodialysis treatment: a consensus statement from the International Society of Renal Nutrition and Metabolism. J Ren Nutr. 2018;28(1):4–12. 29249295 10.1053/j.jrn.2017.10.003

[13] Kalantar-Zadeh K , Ikizler TA. Let them eat during dialysis: an overlooked opportunity to improve outcomes in maintenance hemodialysis patients. J Ren Nutr. 2013;23(3):157–63. 23313434 10.1053/j.jrn.2012.11.001 PMC3632653

[14] Groen BB , Horstman AM, Hamer HM, de Haan M, van Kranenburg J, Bierau J, Poeze M, Wodzig WK, Rasmussen BB, van Loon LJ. Post-prandial protein handling: you are what you just ate. PLoS One. 2015;10(11):e0141582. 26556791 10.1371/journal.pone.0141582 PMC4640549

[15] van Vliet S , Skinner SK, Beals JW, Pagni BA, Fang HY, Ulanov AV, Li Z, Paluska SA, Mazzulla M, West DWD et al. Dysregulated handling of dietary protein and muscle protein synthesis after mixed-meal ingestion in maintenance hemodialysis patients. Kidney Int Rep. 2018;3(6):1403–15. 30450467 10.1016/j.ekir.2018.08.001 PMC6224635

[16] Fuchs CJ , Kouw IWK, Churchward-Venne TA, Smeets JSJ, Senden JM, Lichtenbelt W, Verdijk LB, van Loon LJC. Postexercise cooling impairs muscle protein synthesis rates in recreational athletes. J Physiol. 2020;598(4):755–72. 31788800 10.1113/JP278996 PMC7028023

[17] Sheng K , Zhang P, Chen L, Cheng J, Wu C, Chen J. Intradialytic exercise in hemodialysis patients: a systematic review and meta-analysis. Am J Nephrol. 2014;40(5):478–90. 25504020 10.1159/000368722

[18] Parker K . Intradialytic exercise is medicine for hemodialysis patients. Curr Sports Med Rep. 2016;15(4):269–75. 27399824 10.1249/JSR.0000000000000280

[19] Chung YC , Yeh ML, Liu YM. Effects of intradialytic exercise on the physical function, depression and quality of life for haemodialysis patients: a systematic review and meta-analysis of randomised controlled trials. J Clin Nurs. 2017;26(13–14):1801–13. 27532211 10.1111/jocn.13514

[20] Bennett PN , Breugelmans L, Barnard R, Agius M, Chan D, Fraser D, McNeill L, Potter L. Sustaining a hemodialysis exercise program: a review. Semin Dial. 2010;23(1):62–73. 20331819 10.1111/j.1525-139X.2009.00652.x

[21] McKenna CF , Salvador AF, Hendriks FK, Harris APY, van Loon LJC, Burd NA. Exercising to offset muscle mass loss in hemodialysis patients: the disconnect between intention and intervention. Semin Dial. 2019;32(4):379–85. 30903629 10.1111/sdi.12805

[22] Fang HY , Burrows BT, King AC, Wilund KR. A comparison of intradialytic versus out-of-clinic exercise training programs for hemodialysis patients. Blood Purif. 2020;49(1–2):151–7. 31851985 10.1159/000503772

[23] Ikizler TA , Cano NJ, Franch H, Fouque D, Himmelfarb J, Kalantar-Zadeh K, Kuhlmann MK, Stenvinkel P, TerWee P, Teta D et al. Prevention and treatment of protein energy wasting in chronic kidney disease patients: a consensus statement by the International Society of Renal Nutrition and Metabolism. Kidney Int. 2013;84(6):1096–107. 23698226 10.1038/ki.2013.147

[24] Gamboa JL , Deger SM, Perkins BW, Mambungu C, Sha F, Mason OJ, Stewart TG, Ikizler TA. Effects of long-term intra-dialytic oral nutrition and exercise on muscle protein homeostasis and markers of mitochondrial content in patients on hemodialysis. Am J Physiol Renal Physiol. 2020;319(5):F885–94. 32985237 10.1152/ajprenal.00026.2020 PMC7789984

[25] Borg G . Borg's perceived exertion and pain scales. Champaign (IL): Human Kinetics; 1998.

[26] National Institute of Public Health of the Ministry of Health, Welfare and Sport. Dutch food composition database. [Internet]. [cited 2020 May 1]. Available from: https://nevo-online.rivm.nl/.

[27] Broers NJH , Martens RJH, Cornelis T, van der Sande FM, Diederen NMP, Hermans MMH, Wirtz J, Stifft F, Konings C, Dejagere T et al. Physical activity in end-stage renal disease patients: the effects of starting dialysis in the first 6 months after the transition period. Nephron. 2017;137(1):47–56. 28591752 10.1159/000476072 PMC5872558

[28] Waterval WA , Scheijen JL, Ortmans-Ploemen MM, Habets-van der Poel CD, Bierau J. Quantitative UPLC-MS/MS analysis of underivatised amino acids in body fluids is a reliable tool for the diagnosis and follow-up of patients with inborn errors of metabolism. Clin Chim Acta. 2009;407(1–2):36–42. 19559691 10.1016/j.cca.2009.06.023

[29] Fuchs CJ , Hermans WJH, Holwerda AM, Smeets JSJ, Senden JM, van Kranenburg J, Gijsen AP, Wodzig W, Schierbeek H, Verdijk LB et al. Branched-chain amino acid and branched-chain ketoacid ingestion increases muscle protein synthesis rates in vivo in older adults: a double-blind, randomized trial. Am J Clin Nutr. 2019;110(4):862–72. 31250889 10.1093/ajcn/nqz120 PMC6766442

[30] Pupim LB , Majchrzak KM, Flakoll PJ, Ikizler TA. Intradialytic oral nutrition improves protein homeostasis in chronic hemodialysis patients with deranged nutritional status. J Am Soc Nephrol. 2006;17(11):3149–57. 17021267 10.1681/ASN.2006040413

[31] Lim VS , Ikizler TA, Raj DS, Flanigan MJ. Does hemodialysis increase protein breakdown? Dissociation between whole-body amino acid turnover and regional muscle kinetics. J Am Soc Nephrol. 2005;16(4):862–8. 15716333 10.1681/ASN.2004080624

[32] Raj DS , Zager P, Shah VO, Dominic EA, Adeniyi O, Blandon P, Wolfe R, Ferrando A. Protein turnover and amino acid transport kinetics in end-stage renal disease. Am J Physiol Endocrinol Metab. 2004;286(1):E136–43. 13129859 10.1152/ajpendo.00352.2003

[33] Hendriks FK , Smeets JSJ, van der Sande FM, Kooman JP, van Loon LJC. Dietary protein and physical activity interventions to support muscle maintenance in end-stage renal disease patients on hemodialysis. Nutrients. 2019;11(12):2972. 10.3390/nu11122972 PMC6950262 31817402

[34] Bolasco P . Hemodialysis-nutritional flaws in diagnosis and prescriptions: could amino acid losses be the sharpest “sword of Damocles”?. Nutrients. 2020;12(6):1773. 10.3390/nu12061773 PMC7353226 32545868

[35] Viana JL , Martins P, Parker K, Madero M, Perez Grovas H, Anding K, Degenhardt S, Gabrys I, Raugust S, West C et al. Sustained exercise programs for hemodialysis patients: the characteristics of successful approaches in Portugal, Canada, Mexico, and Germany. Semin Dial. 2019;32(4):320–30. 31087375 10.1111/sdi.12814

[36] Wilund KR , Jeong JH, Greenwood SA. Addressing myths about exercise in hemodialysis patients. Semin Dial. 2019;32(4):297–302. 31025450 10.1111/sdi.12815

[37] Clarkson MJ , Bennett PN, Fraser SF, Warmington SA. Exercise interventions for improving objective physical function in patients with end-stage kidney disease on dialysis: a systematic review and meta-analysis. Am J Physiol Renal Physiol. 2019;316(5):F856–72. 30759022 10.1152/ajprenal.00317.2018

[38] Heiwe S , Jacobson SH. Exercise training in adults with CKD: a systematic review and meta-analysis. Am J Kidney Dis. 2014;64(3):383–93. 24913219 10.1053/j.ajkd.2014.03.020

[39] Kouidi E , Grekas D, Fau-Deligiannis A, Deligiannis A, Fau-Tourkantonis A, Tourkantonis A. Outcomes of long-term exercise training in dialysis patients: comparison of two training programs.Clin Nephrol. 2004;61(Suppl 1):S31–8. 15233245

[40] Lightfoot CJ , Wilkinson TJ, Song Y, Burton JO, Smith AC. Perceptions of exercise benefits and barriers: the influence on physical activity behaviour in individuals undergoing haemodialysis and peritoneal dialysis. J Nephrol[epub ahead of print 26 Mar 2021]. In press. 10.1007/s40620-021-01024-y PMC8610943 33770396

[41] Cheema BS , Smith BC, Singh MA. A rationale for intradialytic exercise training as standard clinical practice in ESRD. Am J Kidney Dis. 2005;45(5):912–6. 15861357 10.1053/j.ajkd.2005.01.030

[42] Salhab N , Alrukhaimi M, Kooman J, Fiaccadori E, Aljubori H, Rizk R, Karavetian M. Effect of intradialytic exercise on hyperphosphatemia and malnutrition. Nutrients. 2019;11(10):2464. 10.3390/nu11102464 PMC6836201 31618888

[43] Kirkman DL , Scott M, Kidd J, Macdonald JH. The effects of intradialytic exercise on hemodialysis adequacy: a systematic review. Semin Dial. 2019;32(4):368–78. 30968465 10.1111/sdi.12785

[44] Jeong JH , Biruete A, Tomayko EJ, Wu PT, Fitschen P, Chung HR, Ali M, McAuley E, Fernhall B, Phillips SA et al. Results from the randomized controlled IHOPE trial suggest no effects of oral protein supplementation and exercise training on physical function in hemodialysis patients. Kidney Int. 2019;96(3):777–86. 31200945 10.1016/j.kint.2019.03.018 PMC6708720

[45] Phillips SM , Tipton KD, Aarsland A, Wolf SE, Wolfe RR. Mixed muscle protein synthesis and breakdown after resistance exercise in humans. Am J Physiol Endocrinol Metab. 1997;273(1):E99–E107. 10.1152/ajpendo.1997.273.1.E99 9252485

[46] Holwerda AM , Paulussen KJM, Overkamp M, Goessens JPB, Kramer IF, Wodzig W, Verdijk LB, van Loon LJC. Dose-dependent increases in whole-body net protein balance and dietary protein-derived amino acid incorporation into myofibrillar protein during recovery from resistance exercise in older men. J Nutr. 2019;149(2):221–30. 30722014 10.1093/jn/nxy263 PMC6374151

[47] Tipton KD , Elliott TA, Cree MG, Wolf SE, Sanford AP, Wolfe RR. Ingestion of casein and whey proteins result in muscle anabolism after resistance exercise. Med Sci Sports Exercise. 2004;36(12):2073–81. 10.1249/01.mss.0000147582.99810.c5 15570142

[48] Hendriks FK , Kooman JP, van Loon LJC. Dietary protein interventions to improve nutritional status in end-stage renal disease patients undergoing hemodialysis. Curr Opin Clin Nutr Metab Care. 2021;24(1):79–87. 33060457 10.1097/MCO.0000000000000703 PMC7752218

[49] Dong J , Sundell MB, Pupim LB, Wu P, Shintani A, Ikizler TA. The effect of resistance exercise to augment long-term benefits of intradialytic oral nutritional supplementation in chronic hemodialysis patients. J Ren Nutr. 2011;21(2):149–59. 20580251 10.1053/j.jrn.2010.03.004 PMC2947559

[50] Oquendo LG , Asencio JMM, de Las Nieves CB. Contributing factors for therapeutic diet adherence in patients receiving haemodialysis treatment: an integrative review. J Clin Nurs. 2017;26(23–24):3893–905. 28295744 10.1111/jocn.13804

