# Supplementary material for: Amino acid removal during hemodialysis can be compensated for by protein ingestion and is not compromised by intradialytic exercise: a randomized controlled crossover trial
Source: Am J Clin Nutr. 2021 Sep 12;114(6):2074–83. doi: 10.1093/ajcn/nqab274 (PMC8634611; doi:10.1093/ajcn/nqab274)
Supplement: nqab274_Supplemental_File [file nqab274_supplemental_file.zip › Supplemental File 1.docx]

**On-line Supplementary Material**

**Supplementary File 1: CONSORT flow diagram**

**Amino acid removal during hemodialysis can be compensated for by protein ingestion and is not affected by intradialytic exercise**

*Floris K. Hendriks, Joey S.J. Smeets, Janneau M.X. van Kranenburg, Natascha J.H. Broers, Frank M. van der Sande, Lex B. Verdijk, Jeroen P. Kooman, and Luc J.C. van Loon.*

**
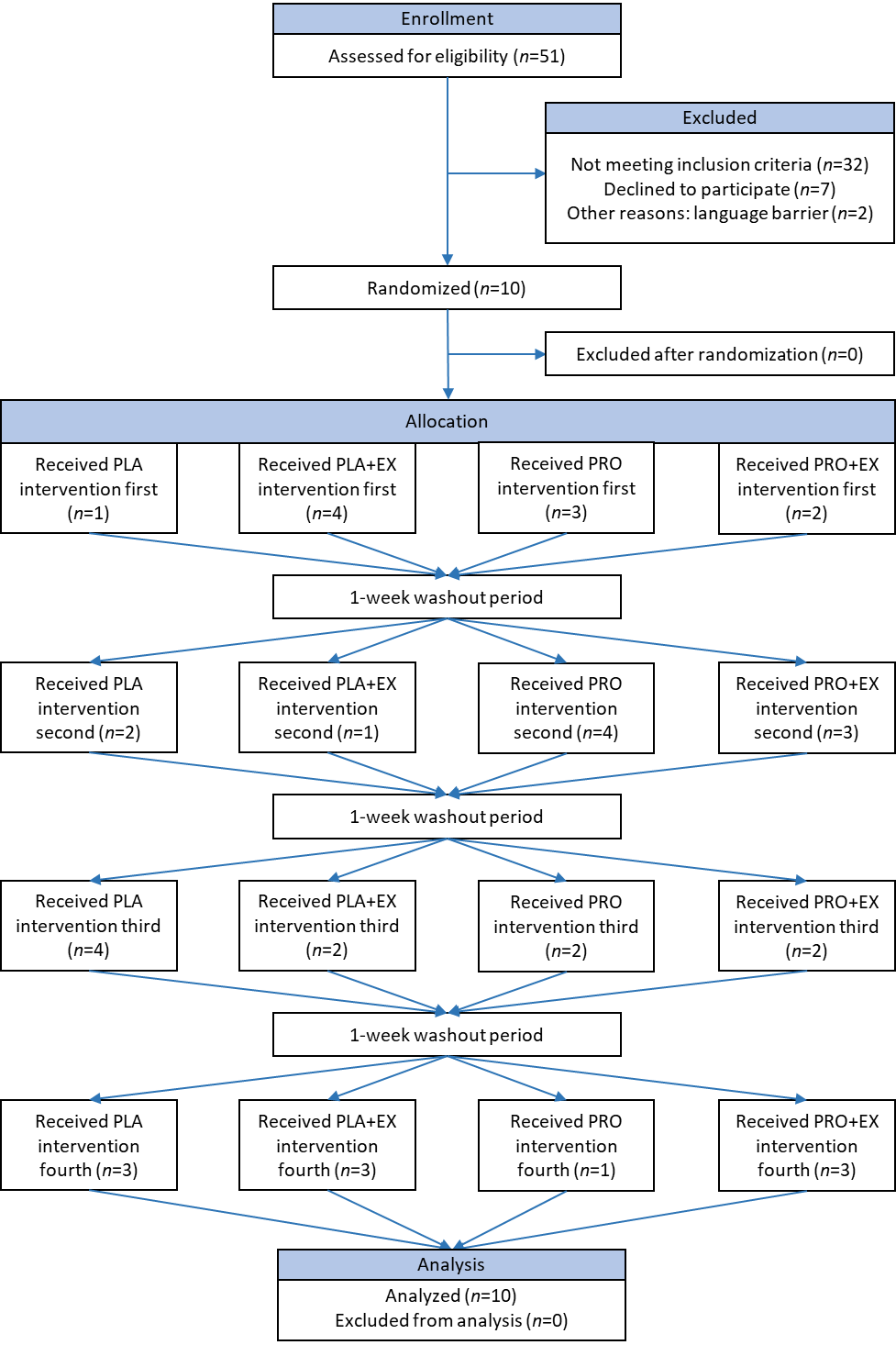

SUPPLEMENTARY FIGURE 1**

**Consolidated Standards of Reporting Trials (CONSORT) flow chart.** PLA, placebo; PLA+EX, placebo and exercise; PRO, protein; PRO+EX, protein and exercise.
