# Supplementary material for: Amino acid removal during hemodialysis can be compensated for by protein ingestion and is not compromised by intradialytic exercise: a randomized controlled crossover trial
Source: Am J Clin Nutr. 2021 Sep 12;114(6):2074–83. doi: 10.1093/ajcn/nqab274 (PMC8634611; doi:10.1093/ajcn/nqab274)
Supplement: nqab274_Supplemental_File [file nqab274_supplemental_file.zip › Supplemental File 2.docx]

**On-line Supplementary Material**

**Supplementary File 2: Individual amino acid figures**

**Amino acid removal during hemodialysis can be compensated for by protein ingestion and is not affected by intradialytic exercise**

*Floris K. Hendriks, Joey S.J. Smeets, Janneau M.X. van Kranenburg, Natascha J.H. Broers, Frank M. van der Sande, Lex B. Verdijk, Jeroen P. Kooman, and Luc J.C. van Loon.*

**TABLE OF CONTENT**

| **On-line Supplementary Material** | **Page number** |
| --- | --- |
| Supplementary statistical analysis for  Supplementary Figures 2 - 21 | 3 |
| *Plasma concentrations, spent dialysate concentrations, incremental area under the curve of plasma concentrations, and removal throughout hemodialysis at rest and following exercise with and without protein ingestion of:* |  |
| Alanine (Supplementary Figure 2) | 4 |
| Arginine (Supplementary Figure 3) | 5 |
| Asparagine (Supplementary Figure 4) | 6 |
| Cysteine (Supplementary Figure 5) | 7 |
| Glutamic acid (Supplementary Figure 6) | 8 |
| Glutamine (Supplementary Figure 7) | 9 |
| Glycine (Supplementary Figure 8) | 10 |
| Histidine (Supplementary Figure 9) | 11 |
| Isoleucine (Supplementary Figure 10) | 12 |
| Leucine (Supplementary Figure 11) | 13 |
| Lysine (Supplementary Figure 12) | 14 |
| Methionine (Supplementary Figure 13) | 15 |
| Ornithine (Supplementary Figure 14) | 16 |
| Phenylalanine (Supplementary Figure 15) | 17 |
| Proline (Supplementary Figure 16) | 18 |
| Serine (Supplementary Figure 17) | 19 |
| Threonine (Supplementary Figure 18) | 20 |
| Tryptophan (Supplementary Figure 19) | 21 |
| Tyrosine (Supplementary Figure 20) | 22 |
| Valine (Supplementary Figure 21) | 23 |

**SUPPLEMENTARY STATISTICAL ANALYSIS FOR**

**SUPPLEMENTARY FIGURES 2 - 21**

Plasma and spent dialysate amino acid concentrations over time were assessed using three-way repeated-measures ANOVA with time, protein ingestion (yes/no) and exercise (yes/no) as within-subject factors. In case of non-sphericity, the Greenhouse-Geisser correction was used. The incremental area under the curve of plasma amino acid concentrations representing the t=0-240 min period and amino acid removal were analyzed by two-way repeated-measures ANOVA with protein ingestion (yes/no) and exercise (yes/no) as within subject variables. In case of non-sphericity, the Greenhouse-Geisser correction was used.

**
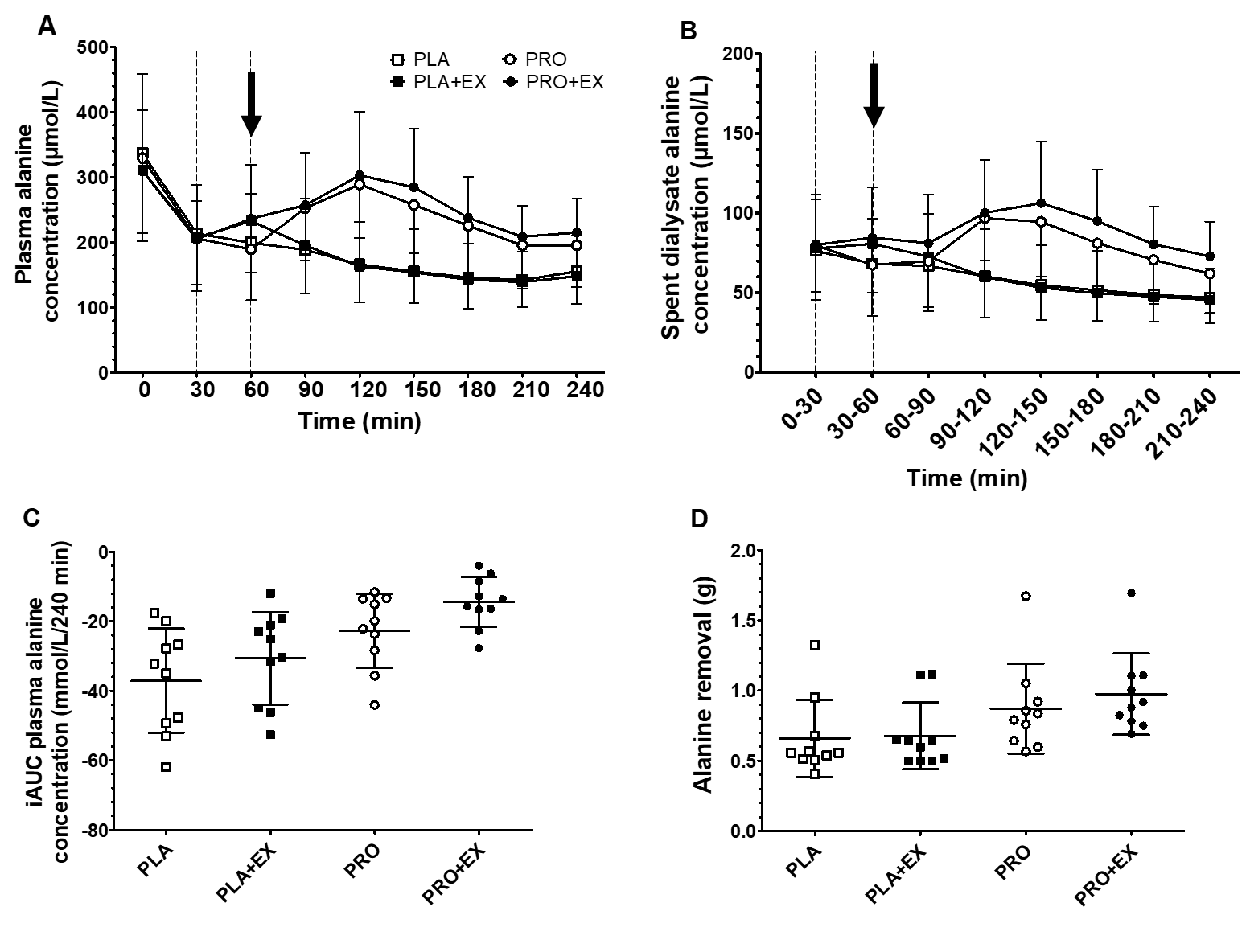
 SUPPLEMENTARY FIGURE 2**

**(A) Plasma alanine concentrations, (B) spent dialysate alanine concentrations, (C) incremental area under the curve of plasma alanine concentrations, and (D) alanine removal** **throughout hemodialysis at rest and following exercise with and without protein ingestion**. The dotted lines represent the start and end of intradialytic exercise and the arrow represents the ingestion of the test beverage. Values, *n*=10 for all, are expressed as means±SDs. **A)** Protein *P*<0.001; Exercise *P*=0.441; Time *P*<0.001; Protein × Exercise *P*=0.053; Protein × Time *P*<0.001; Exercise × Time *P*=0.004; Protein × Exercise × Time *P*=0.575. **B)** Protein *P*<0.001; Exercise *P*=0.078; Time *P*<0.001; Protein × Exercise *P*=0.055; Protein × Time *P*<0.001; Exercise × Time *P*=0.030; Protein × Exercise × Time *P*=0.089. **C)** Protein *P*<0.001; Exercise *P*=0.007; Protein × Exercise *P*=0.693. **D)** Protein *P*<0.001; Exercise *P*=0.087; Protein × Exercise *P*=0.061. iAUC, incremental area under the curve; PLA, placebo; PLA+EX, placebo and exercise; PRO, protein; PRO+EX, protein and exercise.


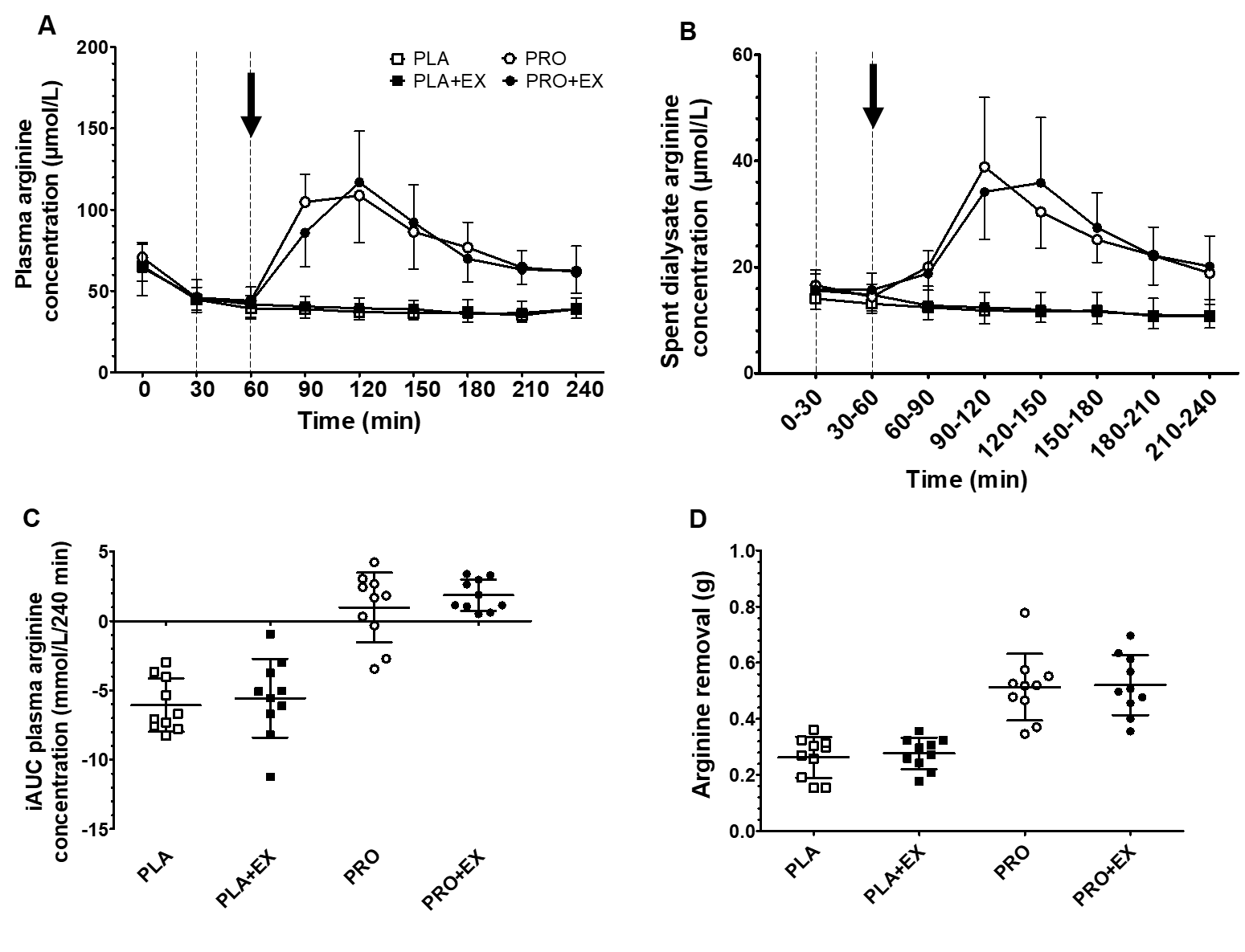


**SUPPLEMENTARY FIGURE 3**

**(A) Plasma arginine concentrations, (B) spent dialysate arginine concentrations, (C) incremental area under the curve of plasma arginine concentrations, and (D) arginine removal** **throughout hemodialysis at rest and following exercise with and without protein ingestion**. The dotted lines represent the start and end of intradialytic exercise and the arrow represents the ingestion of the test beverage. Values, *n*=10 for all, are expressed as means±SDs. **A)** Protein *P*<0.001; Exercise *P*=0.716; Time *P*<0.001; Protein × Exercise *P*=0.315; Protein × Time *P*<0.001; Exercise × Time *P=*0.040; Protein × Exercise × Time *P*=0.036. **B)** Protein *P*<0.001; Exercise *P*=0.538; Time *P*<0.001; Protein × Exercise *P*=0.896; Protein × Time *P*<0.001; Exercise × Time *P*=0.151; Protein × Exercise × Time *P*=0.121. **C)** Protein *P*<0.001; Exercise *P*=0.265; Protein × Exercise *P*=0.745. **D)** Protein *P*<0.001; Exercise *P*=0.546; Protein × Exercise *P*=0.792. iAUC, incremental area under the curve; PLA, placebo; PLA+EX, placebo and exercise; PRO, protein; PRO+EX, protein and exercise.


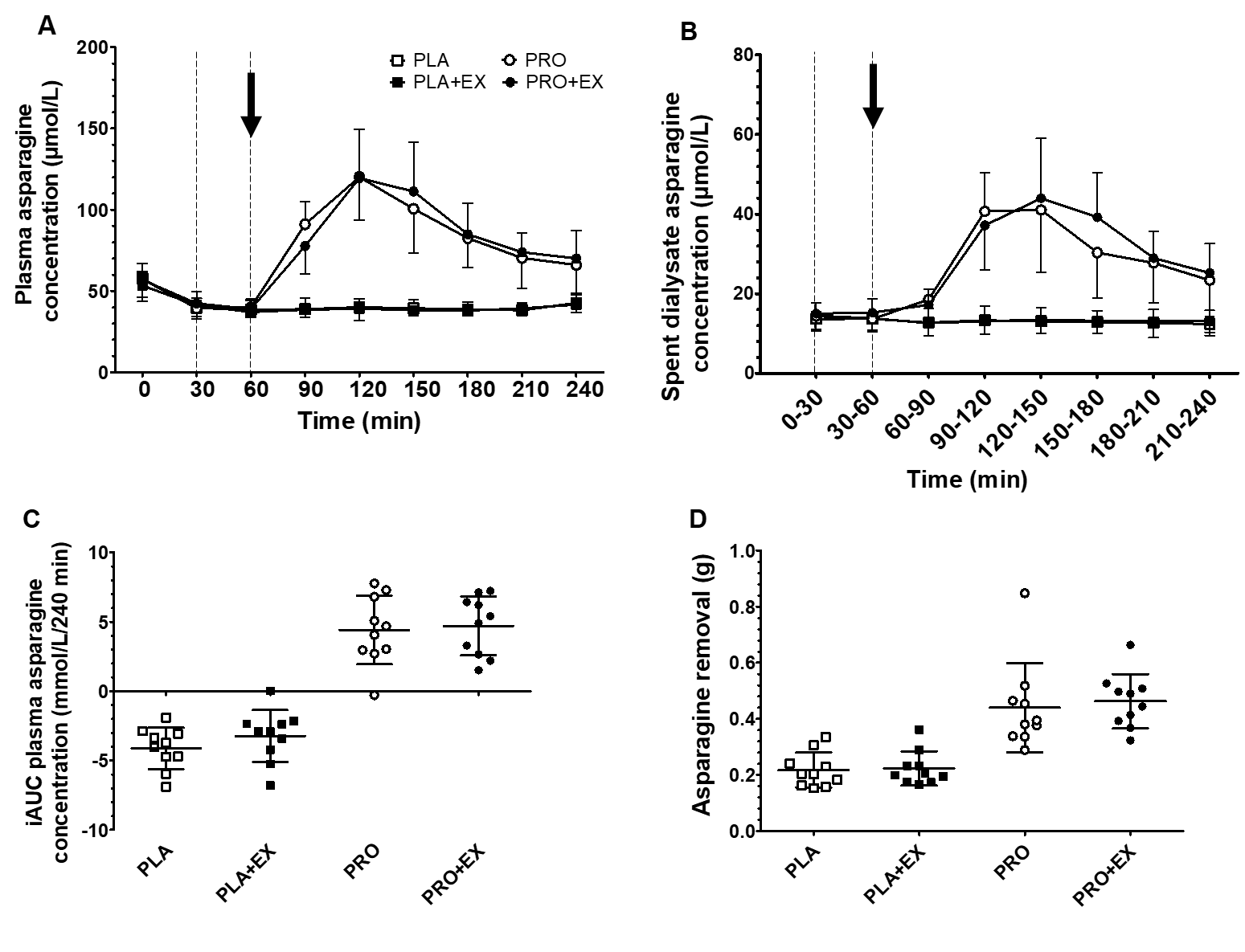


**SUPPLEMENTARY FIGURE 4**

**(A) Plasma asparagine concentrations, (B) spent dialysate asparagine concentrations, (C) incremental area under the curve of plasma asparagine concentrations, and (D) asparagine removal** **throughout hemodialysis at rest and following exercise with and without protein ingestion**. The dotted lines represent the start and end of intradialytic exercise and the arrow represents the ingestion of the test beverage. Values, *n*=10 for all, are expressed as means±SDs. **A)** Protein *P*<0.001; Exercise *P*=0.950; Time *P*<0.001; Protein × Exercise *P*=0.426; Protein × Time *P*<0.001; Exercise × Time *P*=0.142; Protein × Exercise × Time *P*=0.274. **B)** Protein *P*<0.001; Exercise *P*=0.418; Time *P*<0.001; Protein × Exercise *P*=0.454; Protein × Time *P*<0.001; Exercise × Time *P*=0.089; Protein × Exercise × Time *P*=0.174. **C)** Protein *P*<0.001; Exercise *P*=0.322; Protein × Exercise *P*=0.561. **D)** Protein *P*<0.001; Exercise *P*=0.458; Protein × Exercise *P*=0.563. iAUC, incremental area under the curve; PLA, placebo; PLA+EX, placebo and exercise; PRO, protein; PRO+EX, protein and exercise.


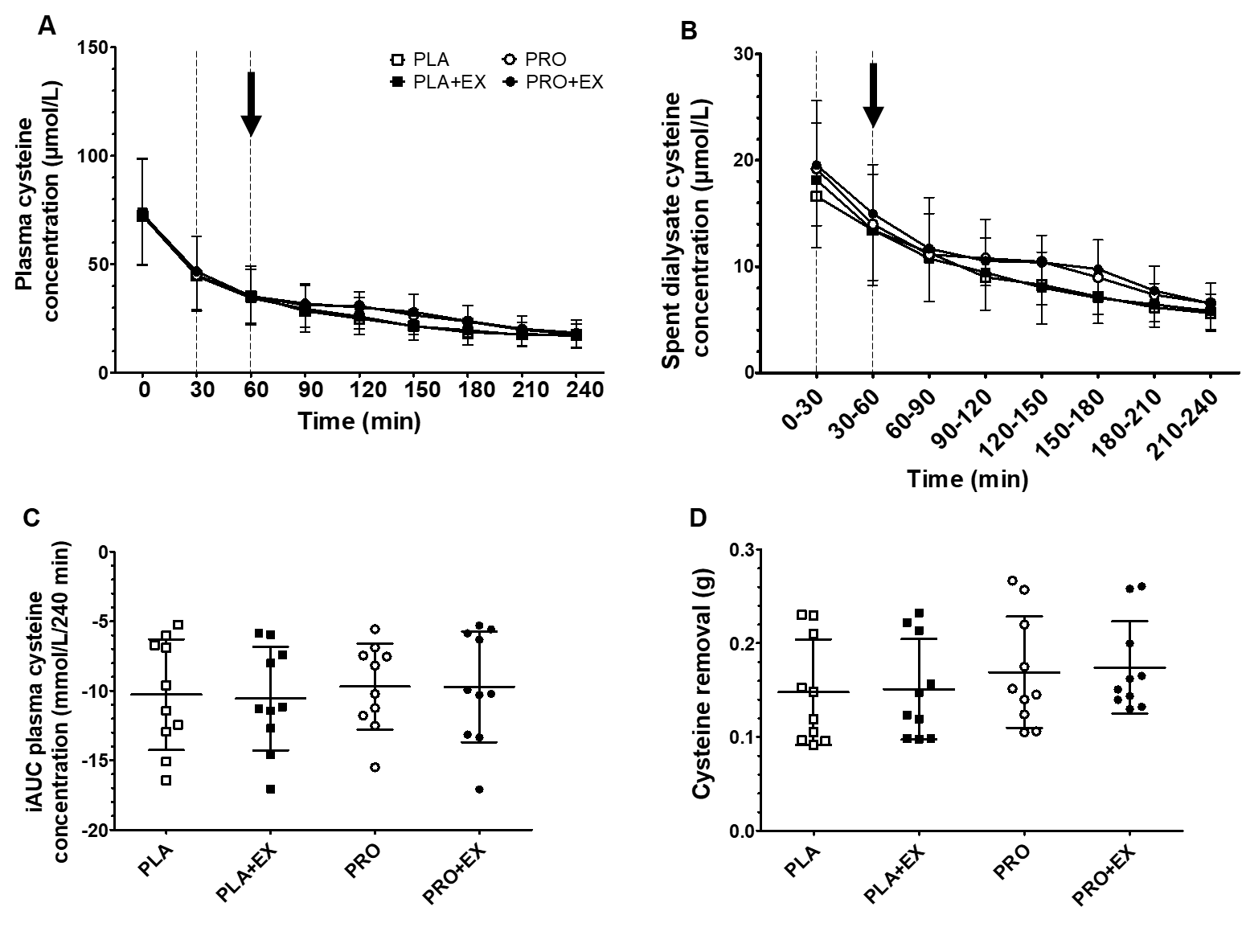


**SUPPLEMENTARY FIGURE 5**

**(A) Plasma cysteine concentrations, (B) spent dialysate cysteine concentrations, (C) incremental area under the curve of plasma cysteine concentrations, and (D) cysteine removal** **throughout hemodialysis at rest and following exercise with and without protein ingestion**. The dotted lines represent the start and end of intradialytic exercise and the arrow represents the ingestion of the test beverage. Values, *n*=10 for all, are expressed as means±SDs. **A)** Protein *P*=0.041; Exercise *P*=0.434; Time *P*<0.001; Protein × Exercise *P*=0.816; Protein × Time *P*=0.004; Exercise × Time *P*=0.764; Protein × Exercise × Time *P*=0.724. **B)** Protein *P*=0.007; Exercise *P*=0.349; Time *P*<0.001; Protein × Exercise *P*=0.724; Protein × Time *P*=0.020; Exercise × Time *P*=0.251; Protein × Exercise × Time *P*=0.296. **C)** Protein *P*=0.079; Exercise *P*=0.687; Protein × Exercise *P*=0.651. **D)** Protein *P*=0.007; Exercise *P*=0.333; Protein × Exercise *P*=0.743. iAUC, incremental area under the curve; PLA, placebo; PLA+EX, placebo and exercise; PRO, protein; PRO+EX, protein and exercise.


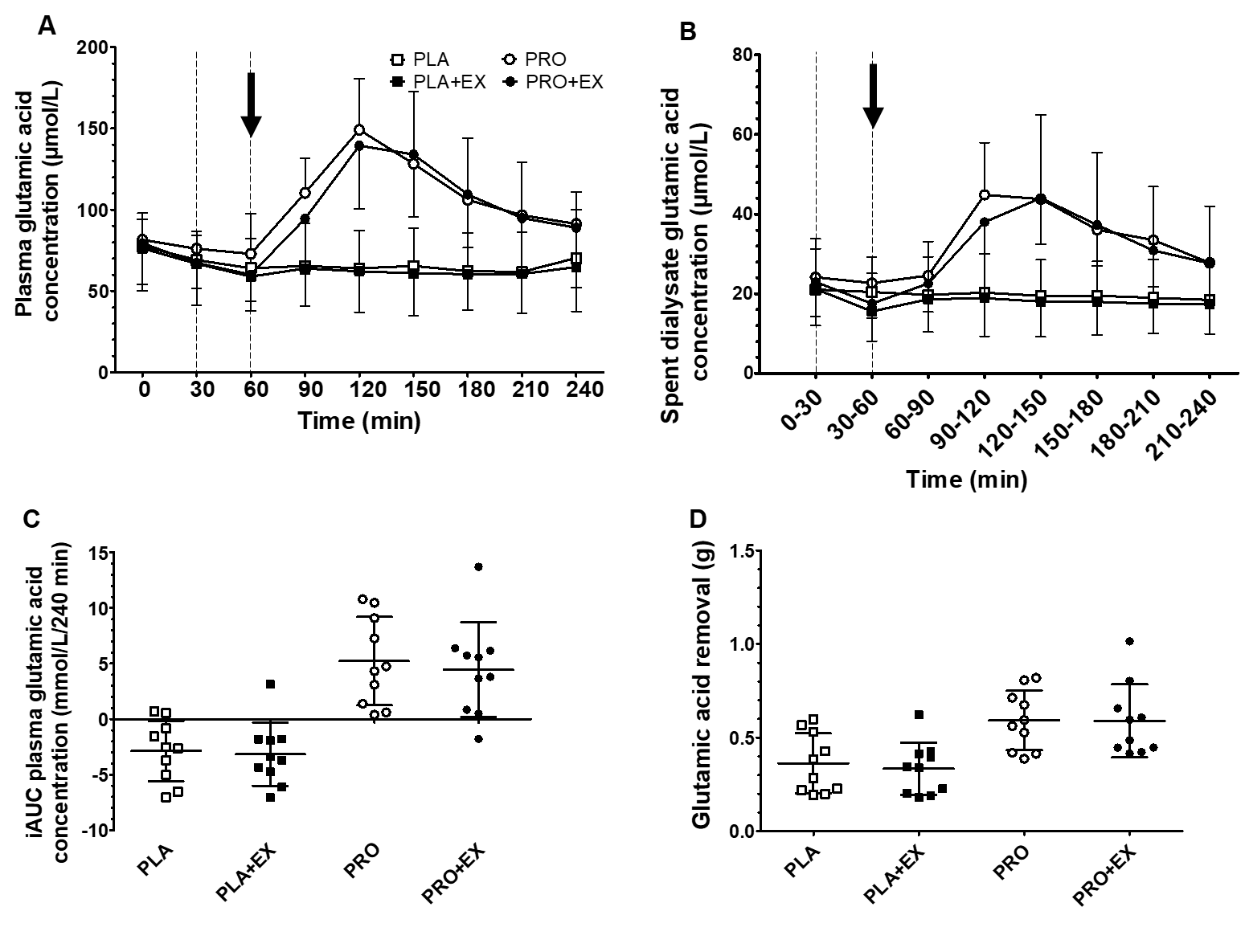


**SUPPLEMENTARY FIGURE 6**

**(A) Plasma glutamic acid concentrations, (B) spent dialysate glutamic acid concentrations, (C) incremental area under the curve of plasma glutamic acid concentrations, and (D) glutamic acid removal** **throughout hemodialysis at rest and following exercise with and without protein ingestion**. The dotted lines represent the start and end of intradialytic exercise and the arrow represents the ingestion of the test beverage. Values, *n*=10 for all, are expressed as means±SDs. **A)** Protein *P*<0.001; Exercise *P*=0.026; Time *P*<0.001; Protein × Exercise *P*=0.703; Protein × Time *P*<0.001; Exercise × Time *P*=0.181; Protein × Exercise × Time *P*=0.451. **B)** Protein *P*<0.001; Exercise *P*=0.275; Time *P*<0.001; Protein × Exercise *P*=0.910; Protein × Time *P*<0.001; Exercise × Time *P*=0.122; Protein × Exercise × Time *P*=0.478. **C)** Protein *P*<0.001; Exercise *P*=0.369; Protein × Exercise *P*=0.840. **D)** Protein *P*<0.001; Exercise *P*=0.384; Protein × Exercise *P*=0.678. iAUC, incremental area under the curve; PLA, placebo; PLA+EX, placebo and exercise; PRO, protein; PRO+EX, protein and exercise.


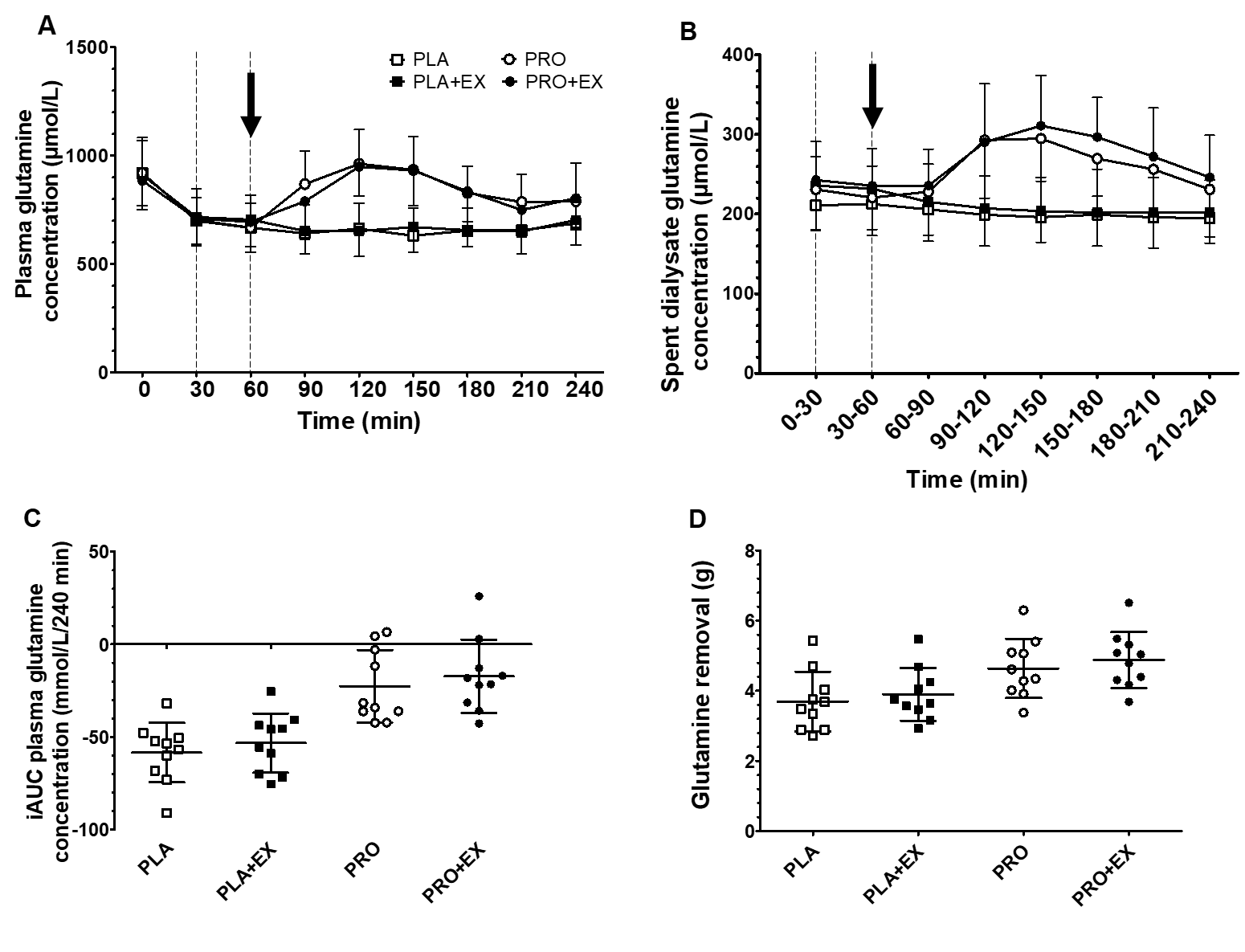


**SUPPLEMENTARY FIGURE 7**

**(A) Plasma glutamine concentrations, (B) spent dialysate glutamine concentrations, (C) incremental area under the curve of plasma glutamine concentrations, and (D) glutamine removal** **throughout hemodialysis at rest and following exercise with and without protein ingestion**. The dotted lines represent the start and end of intradialytic exercise and the arrow represents the ingestion of the test beverage. Values, *n*=10 for all, are expressed as means±SDs. **A)** Protein *P*<0.001; Exercise *P*=0.924; Time *P*<0.001; Protein × Exercise *P*=0.218; Protein × Time *P*<0.001; Exercise × Time *P*=0.103; Protein × Exercise × Time *P*=0.475. **B)** Protein *P*<0.001; Exercise *P*=0.112; Time *P*<0.001; Protein × Exercise *P*=0.772; Protein × Time *P*<0.001; Exercise × Time *P*=0.546; Protein × Exercise × Time *P*=0.078. **C)** Protein *P*<0.001; Exercise *P*=0.309; Protein × Exercise *P*=0.982. **D)** Protein *P*<0.001; Exercise *P*=0.106; Protein × Exercise *P*=0.840. iAUC, incremental area under the curve; PLA, placebo; PLA+EX, placebo and exercise; PRO, protein; PRO+EX, protein and exercise.


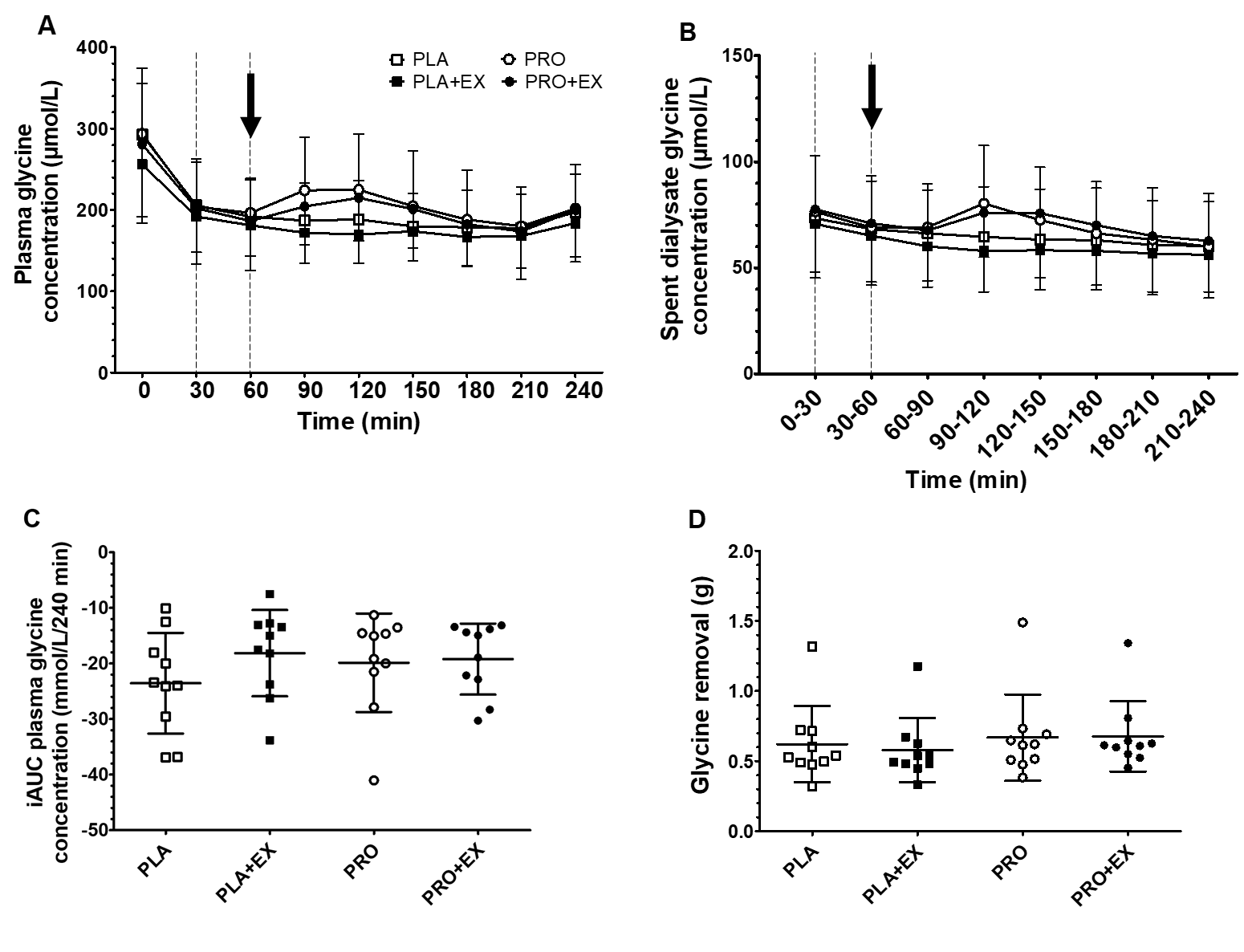


**SUPPLEMENTARY FIGURE 8**

**(A) Plasma glycine concentrations, (B) spent dialysate glycine concentrations, (C) incremental area under the curve of plasma glycine concentrations, and (D) glycine removal** **throughout hemodialysis at rest and following exercise with and without protein ingestion**. The dotted lines represent the start and end of intradialytic exercise and the arrow represents the ingestion of the test beverage. Values, *n*=10 for all, are expressed as means±SDs. **A)** Protein *P*=0.74; Exercise *P*=0.069; Time *P*<0.001; Protein × Exercise *P*=0.318; Protein × Time *P*<0.001; Exercise × Time *P*=0.004; Protein × Exercise × Time *P*=0.148. **B)** Protein *P*=0.013; Exercise *P*=0.446; Time *P*<0.001; Protein × Exercise *P*=0.075; Protein × Time *P*<0.001; Exercise × Time *P*=0.029; Protein × Exercise × Time *P*=0.398. **C)** Protein *P*=0.380; Exercise *P*=0.040; Protein × Exercise *P*=0.033. **D)** Protein *P*=0.013; Exercise *P*=0.451; Protein × Exercise *P*=0.089. iAUC, incremental area under the curve; PLA, placebo; PLA+EX, placebo and exercise; PRO, protein; PRO+EX, protein and exercise.


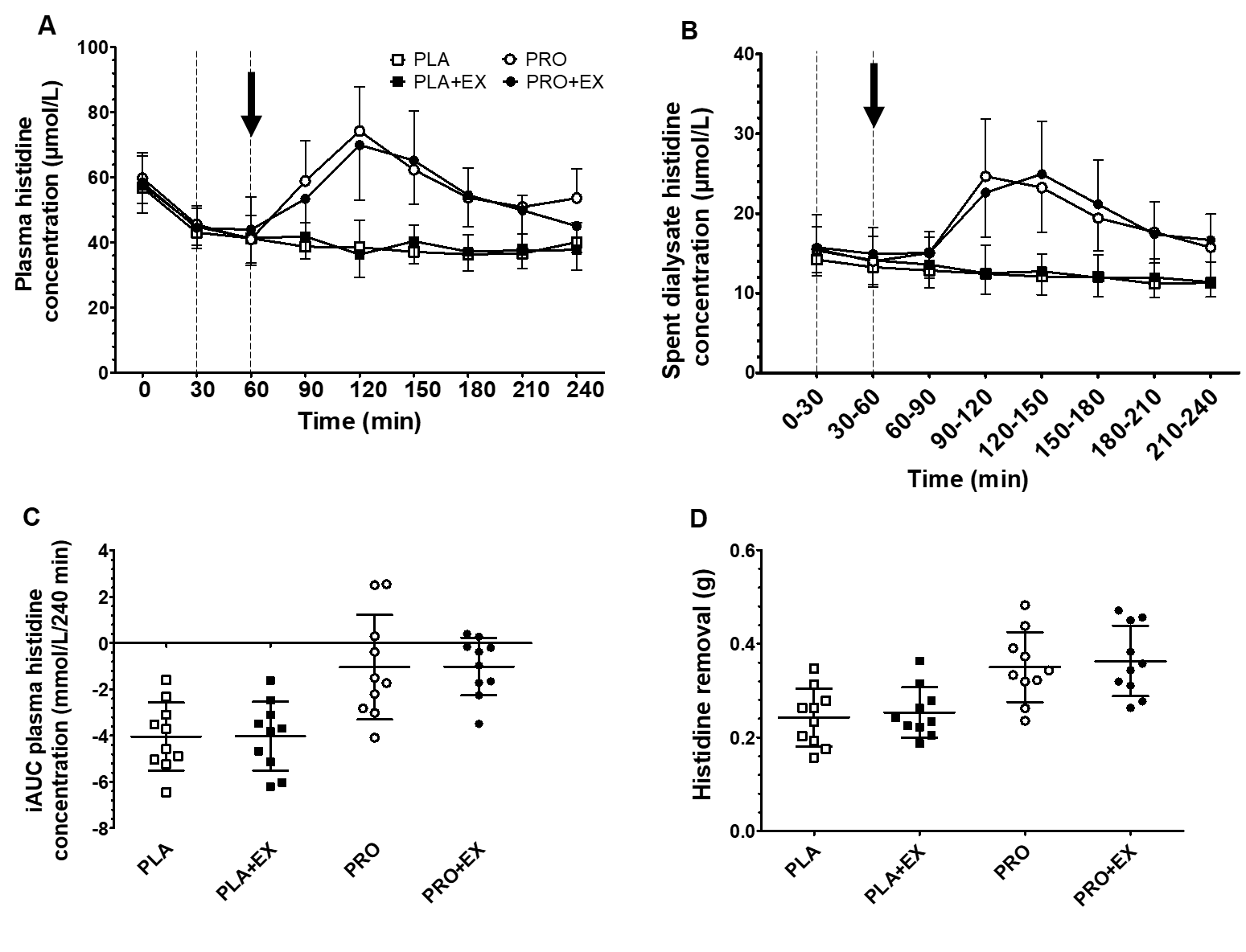


**SUPPLEMENTARY FIGURE 9**

**(A) Plasma histidine concentrations, (B) spent dialysate histidine concentrations, (C) incremental area under the curve of plasma histidine concentrations, and (D) histidine removal** **throughout hemodialysis at rest and following exercise with and without protein ingestion**. The dotted lines represent the start and end of intradialytic exercise and the arrow represents the ingestion of the test beverage. Values, *n*=10 for all, are expressed as means±SDs. **A)** Protein *P*<0.001; Exercise *P*=0.610; Time *P*<0.001; Protein × Exercise *P*=0.291; Protein × Time *P*<0.001; Exercise × Time *P*=0.201; Protein × Exercise × Time *P*=0.537. **B)** Protein *P*<0.001; Exercise *P*=0.490; Time *P*<0.001; Protein × Exercise *P*=0.870; Protein × Time *P*<0.001; Exercise × Time *P*=0.285; Protein × Exercise × Time *P*=0.293. **C)** Protein *P*=0.002; Exercise *P*=0.382; Protein × Exercise *P*=0.240. **D)** Protein *P*<0.001; Exercise *P*=0.384; Protein × Exercise *P*=0.873. iAUC, incremental area under the curve; PLA, placebo; PLA+EX, placebo and exercise; PRO, protein; PRO+EX, protein and exercise.


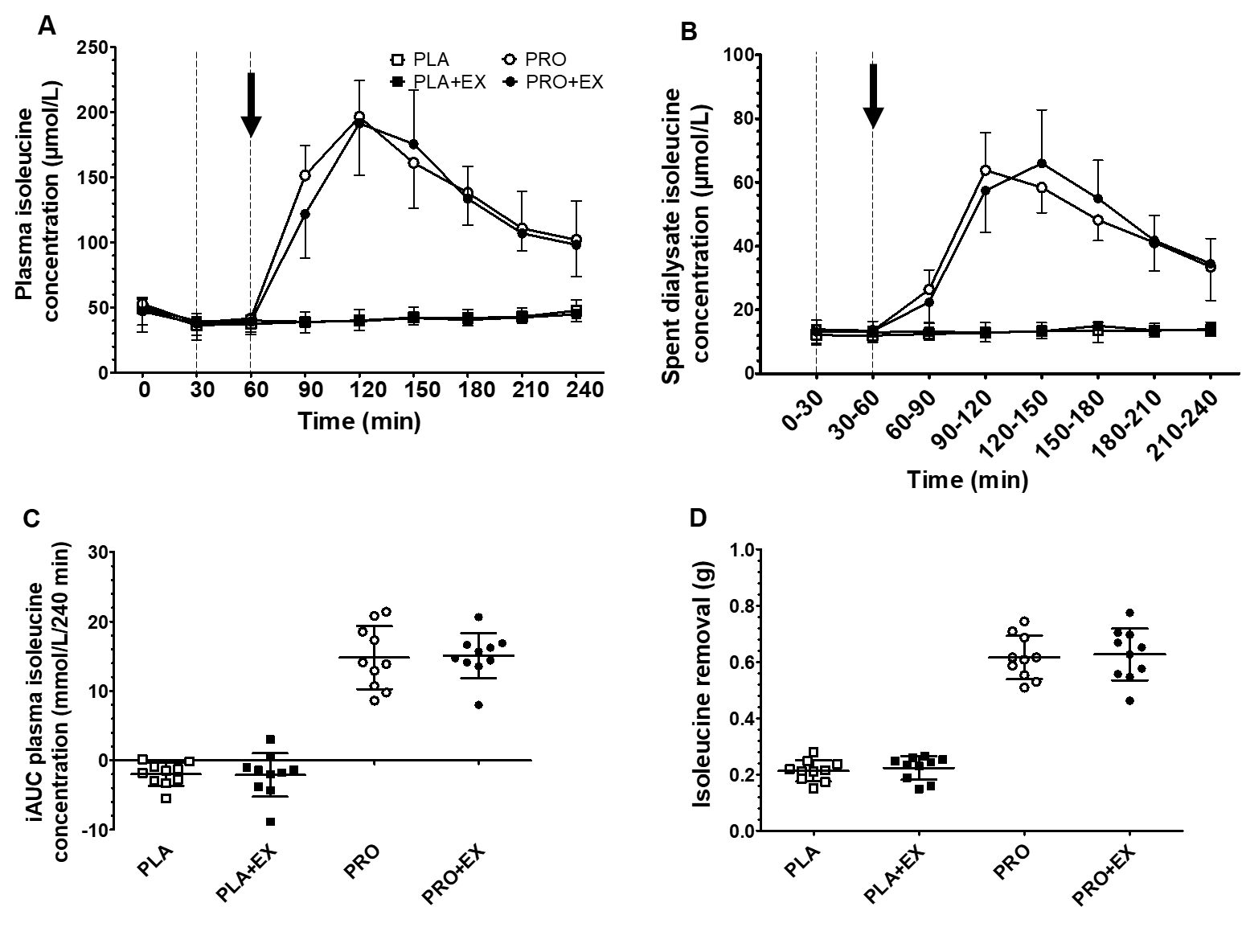


**SUPPLEMENTARY FIGURE 10**

**(A) Plasma isoleucine concentrations, (B) spent dialysate isoleucine concentrations, (C) incremental area under the curve of plasma isoleucine concentrations, and (D) isoleucine removal** **throughout hemodialysis at rest and following exercise with and without protein ingestion**. The dotted lines represent the start and end of intradialytic exercise and the arrow represents the ingestion of the test beverage. Values, *n*=10 for all, are expressed as means±SDs. **A)** Protein *P*<0.001; Exercise *P*=0.207; Time *P*<0.001; Protein × Exercise *P*=0.083; Protein × Time *P*<0.001; Exercise × Time *P*=0.132; Protein × Exercise × Time *P*=0.172. **B)** Protein *P*<0.001; Exercise *P*=0.625; Time *P*<0.001; Protein × Exercise *P*=0.984; Protein × Time *P*<0.001; Exercise × Time *P*=0.069; Protein × Exercise × Time *P*=0.097. **C)** Protein *P*<0.001; Exercise *P*<0.001; Protein × Exercise *P*=0.196. **D)** Protein *P*<0.001; Exercise *P*=0.592; Protein × Exercise *P*=0.997. iAUC, incremental area under the curve; PLA, placebo; PLA+EX, placebo and exercise; PRO, protein; PRO+EX, protein and exercise.


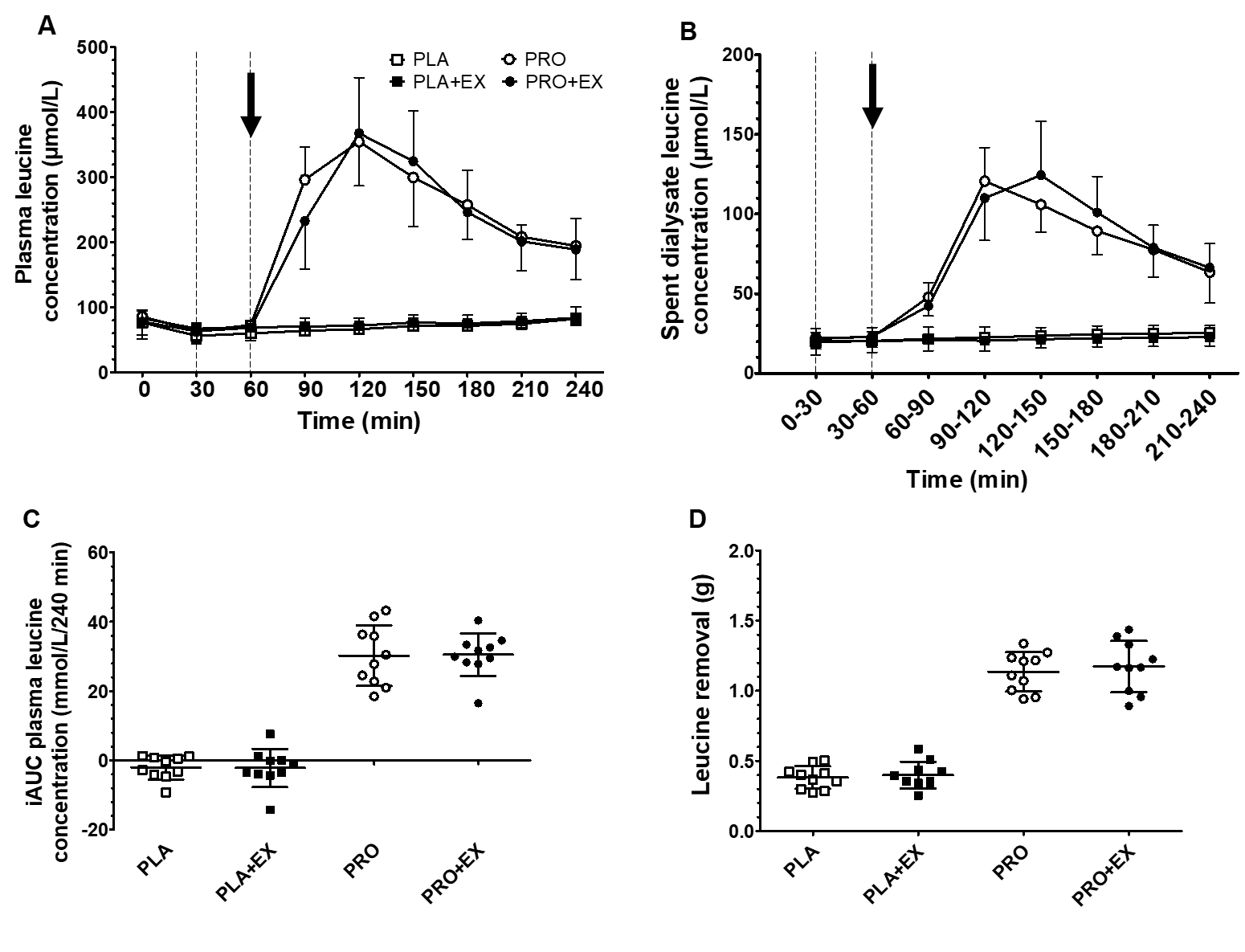


**SUPPLEMENTARY FIGURE 11**

**(A) Plasma leucine concentrations, (B) spent dialysate leucine concentrations, (C) incremental area under the curve of plasma leucine concentrations, and (D) leucine removal** **throughout hemodialysis at rest and following exercise with and without protein ingestion**. The dotted lines represent the start and end of intradialytic exercise and the arrow represents the ingestion of the test beverage. Values, *n*=10 for all, are expressed as means±SDs. **A)** Protein *P*<0.001; Exercise *P*=0.913; Time *P*<0.001; Protein × Exercise *P*=0.041; Protein × Time *P*<0.001; Exercise × Time *P*=0.049; Protein × Exercise × Time *P*=0.151. **B)** Protein *P*<0.001; Exercise *P*=0.854; Time *P*<0.001; Protein × Exercise *P*=0.385; Protein × Time *P*<0.001; Exercise × Time *P*=0.102; Protein × Exercise × Time *P*=0.091. **C)** Protein *P*<0.001; Exercise *P*=0.954; Protein × Exercise *P*=0.913. **D)** Protein *P*<0.001; Exercise *P*=0.449; Protein × Exercise *P*=0.748. iAUC, incremental area under the curve; PLA, placebo; PLA+EX, placebo and exercise; PRO, protein; PRO+EX, protein and exercise.

**
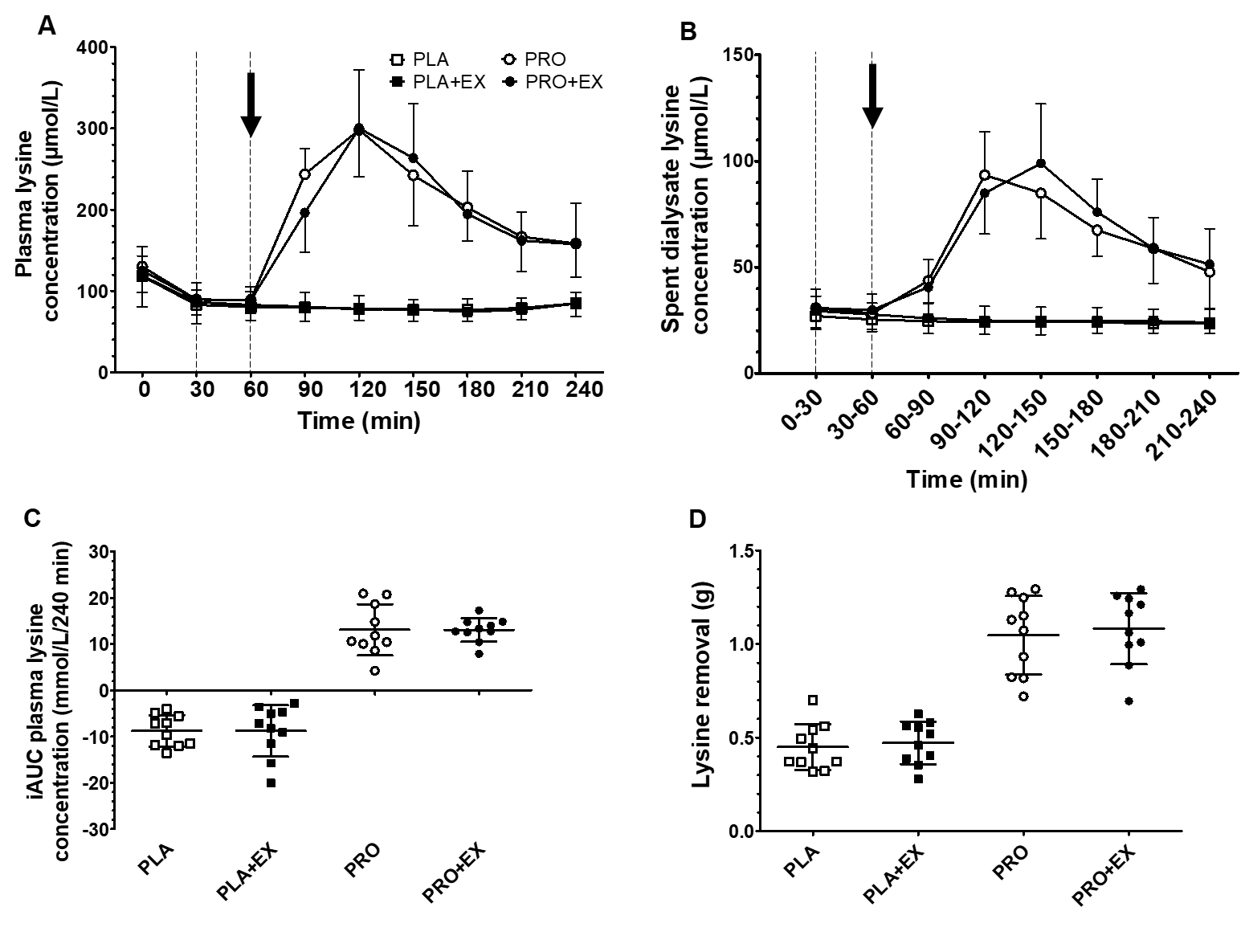
**

**SUPPLEMENTARY FIGURE 12**

**(A) Plasma lysine concentrations, (B) spent dialysate lysine concentrations, (C) incremental area under the curve of plasma lysine concentrations, and (D) lysine removal** **throughout hemodialysis at rest and following exercise with and without protein ingestion**. The dotted lines represent the start and end of intradialytic exercise and the arrow represents the ingestion of the test beverage. Values, *n*=10 for all, are expressed as means±SDs. **A)** Protein *P*<0.001; Exercise *P*=0.495; Time *P*<0.001; Protein × Exercise *P*=0.278; Protein × Time *P*<0.001; Exercise × Time *P*=0.124; Protein × Exercise × Time *P*=0.132. **B)** Protein *P*<0.001; Exercise *P*=0.390; Time *P*<0.001; Protein × Exercise *P*=0.759; Protein × Time *P*<0.001; Exercise × Time *P*=0.141; Protein × Exercise × Time *P*=0.112. **C)** Protein *P*<0.001; Exercise *P*=0.954; Protein × Exercise *P*=0.913. **D)** Protein *P*<0.001; Exercise *P*=0.379; Protein × Exercise *P*=0.820. iAUC, incremental area under the curve; PLA, placebo; PLA+EX, placebo and exercise; PRO, protein; PRO+EX, protein and exercise.


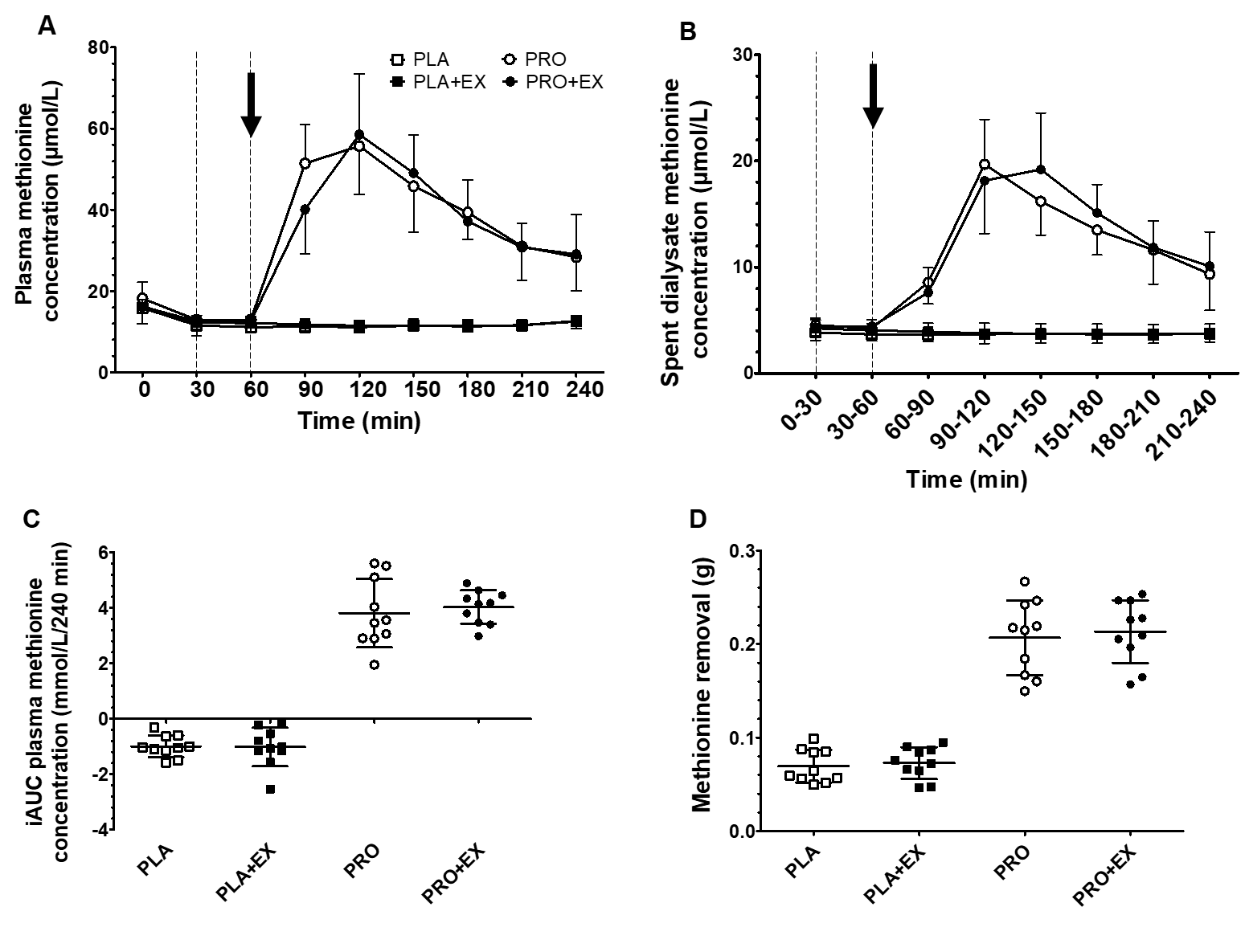


**SUPPLEMENTARY FIGURE 13**

**(A) Plasma methionine concentrations, (B) spent dialysate methionine concentrations, (C) incremental area under the curve of plasma methionine concentrations, and (D) methionine removal** **throughout hemodialysis at rest and following exercise with and without protein ingestion**. The dotted lines represent the start and end of intradialytic exercise and the arrow represents the ingestion of the test beverage. Values, *n*=10 for all, are expressed as means±SDs. **A)** Protein *P*<0.001; Exercise *P*=0.409; Time *P*<0.001; Protein × Exercise *P*=0.141; Protein × Time *P*<0.001; Exercise × Time *P*=0.069; Protein × Exercise × Time *P*=0.0.075. **B)** Protein *P*<0.001; Exercise *P*=0.377; Time *P*<0.001; Protein × Exercise *P*=0.669; Protein × Time *P*<0.001; Exercise × Time *P*=0.141; Protein × Exercise × Time *P*=0.117. **C)** Protein *P*<0.001; Exercise *P*=0.586; Protein × Exercise *P*=0.618. **D)** Protein *P*<0.001; Exercise *P*=0.386; Protein × Exercise *P*=0.752. iAUC, incremental area under the curve; PLA, placebo; PLA+EX, placebo and exercise; PRO, protein; PRO+EX, protein and exercise.


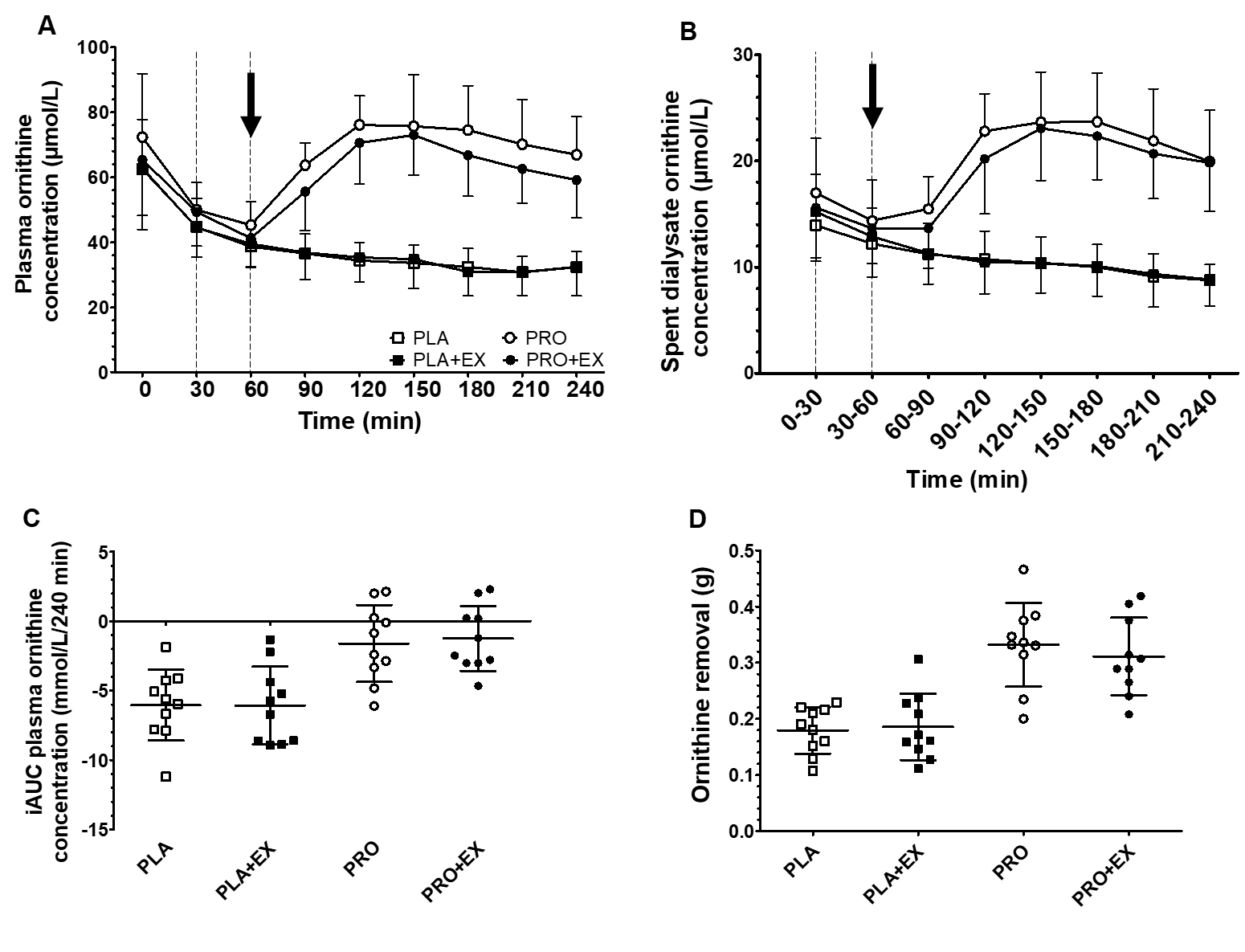


**SUPPLEMENTARY FIGURE 14**

**(A) Plasma ornithine concentrations, (B) spent dialysate ornithine concentrations, (C) incremental area under the curve of plasma ornithine concentrations, and (D) ornithine removal** **throughout hemodialysis at rest and following exercise with and without protein ingestion**. The dotted lines represent the start and end of intradialytic exercise and the arrow represents the ingestion of the test beverage. Values, *n*=10 for all, are expressed as means±SDs. **A)** Protein *P*<0.001; Exercise *P*=0.081; Time *P*<0.001; Protein × Exercise *P*=0.065; Protein × Time *P*<0.001; Exercise × Time *P*=0.562; Protein × Exercise × Time *P*=0.605. **B)** Protein *P*<0.001; Exercise *P*=0.461; Time *P*<0.001; Protein × Exercise *P*=0.100; Protein × Time *P*<0.001; Exercise × Time *P*=0.389; Protein × Exercise × Time *P*=0.499. **C)** Protein *P*<0.001; Exercise *P*=0.747; Protein × Exercise *P*=0.696. **D)** Protein *P*<0.001; Exercise *P*=0.497; Protein × Exercise *P*=0.106. iAUC, incremental area under the curve; PLA, placebo; PLA+EX, placebo and exercise; PRO, protein; PRO+EX, protein and exercise.

**
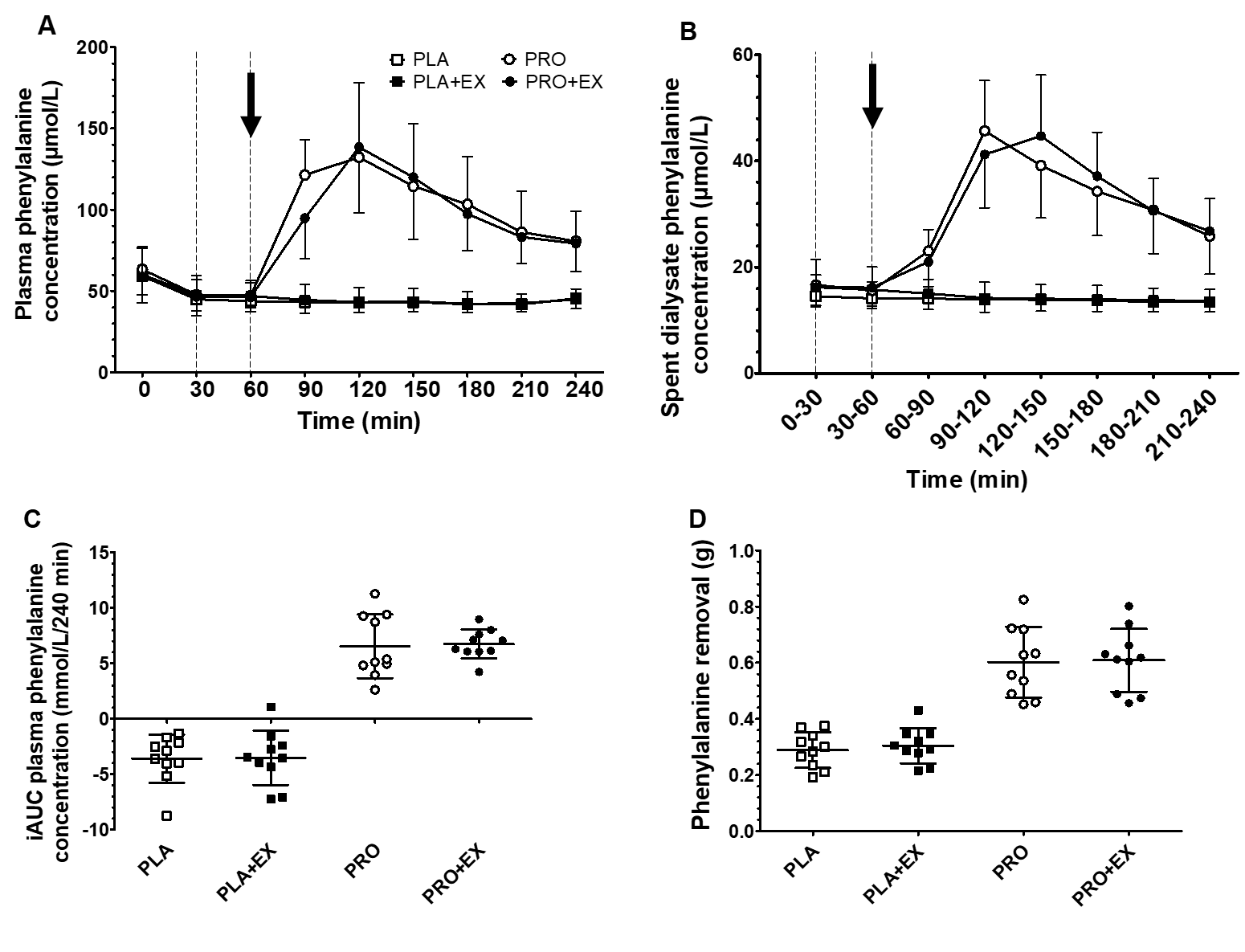
**

**SUPPLEMENTARY FIGURE 15**

**(A) Plasma phenylalanine concentrations, (B) spent dialysate phenylalanine concentrations, (C) incremental area under the curve of plasma phenylalanine concentrations, and (D) phenylalanine removal** **throughout hemodialysis at rest and following exercise with and without protein ingestion**. The dotted lines represent the start and end of intradialytic exercise and the arrow represents the ingestion of the test beverage. Values, *n*=10 for all, are expressed as means±SDs. **A)** Protein *P*<0.001; Exercise *P*=0.251; Time *P*<0.001; Protein × Exercise *P*=0.146; Protein × Time *P*<0.001; Exercise × Time *P*<=0.087; Protein × Exercise × Time *P*=0.104. **B)** Protein *P*<0.001; Exercise *P*=0.450; Time *P*<0.001; Protein × Exercise *P*=0.739; Protein × Time *P*<0.001; Exercise × Time *P*=0.092; Protein × Exercise × Time *P*=0.083. **C)** Protein *P*<0.001; Exercise *P*=0.702; Protein × Exercise *P*=0.917. **D)** Protein *P*<0.001; Exercise *P*=0.440; Protein × Exercise *P*=0.664. iAUC, incremental area under the curve; PLA, placebo; PLA+EX, placebo and exercise; PRO, protein; PRO+EX, protein and exercise.


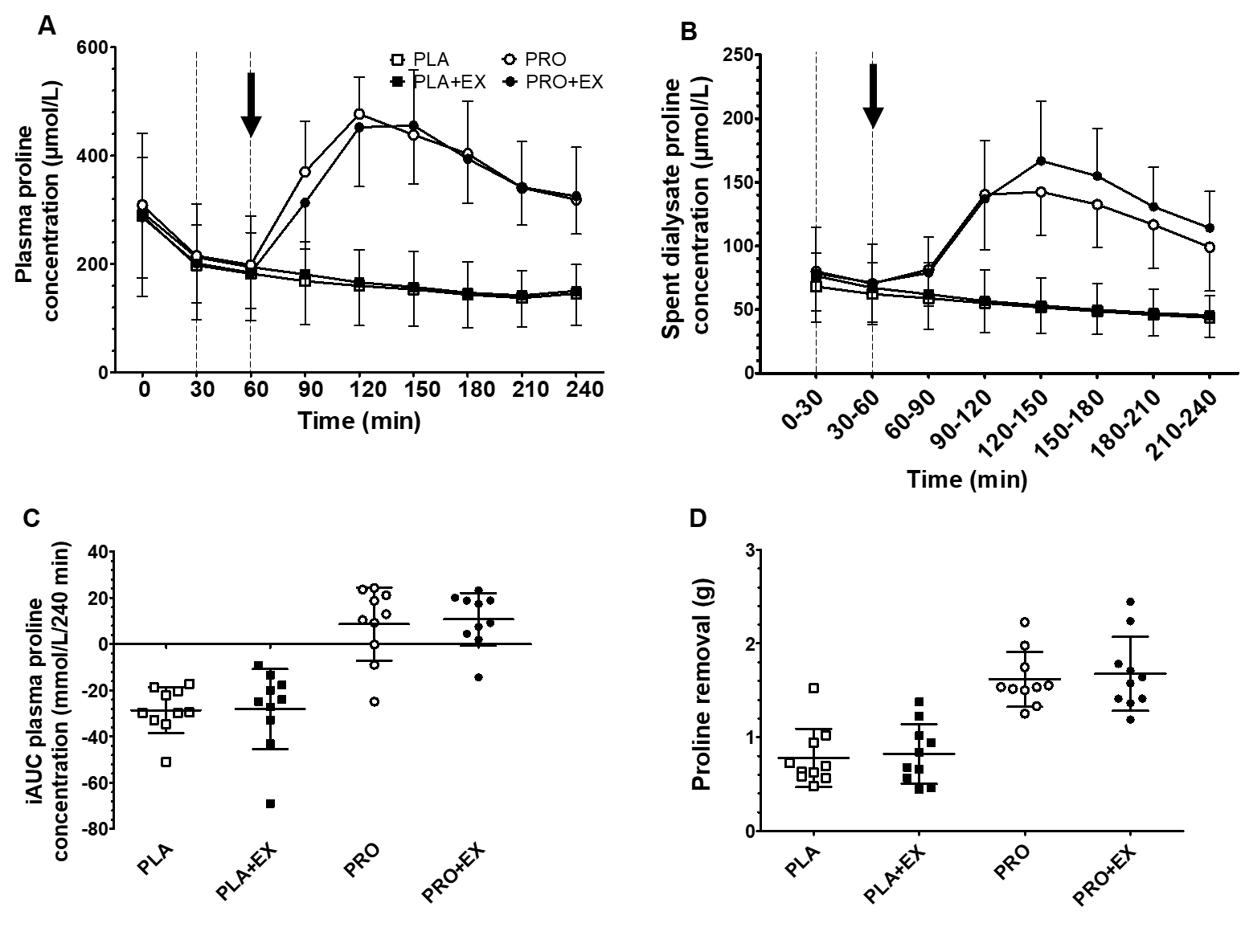


**SUPPLEMENTARY FIGURE 16**

**(A) Plasma proline concentrations, (B) spent dialysate proline concentrations, (C) incremental area under the curve of plasma proline concentrations, and (D) proline removal** **throughout hemodialysis at rest and following exercise with and without protein ingestion**. The dotted lines represent the start and end of intradialytic exercise and the arrow represents the ingestion of the test beverage. Values, *n*=10 for all, are expressed as means±SDs. **A)** Protein *P*<0.001; Exercise *P*=0.738; Time *P*<0.001; Protein × Exercise *P*=0.439; Protein × Time *P*<0.001; Exercise × Time *P*=0.324; Protein × Exercise × Time *P*=0.284. **B)** Protein *P*<0.001; Exercise *P*=0.273; Time *P*<0.001; Protein × Exercise *P*=0.265; Protein × Time *P*<0.001; Exercise × Time *P*=0.013; Protein × Exercise × Time *P*=0.026. **C)** Protein *P*<0.001; Exercise *P*=0.559; Protein × Exercise *P*=0.830. **D)** Protein *P*<0.001; Exercise *P*=0.485; Protein × Exercise *P*=0.749. iAUC, incremental area under the curve; PLA, placebo; PLA+EX, placebo and exercise; PRO, protein; PRO+EX, protein and exercise.


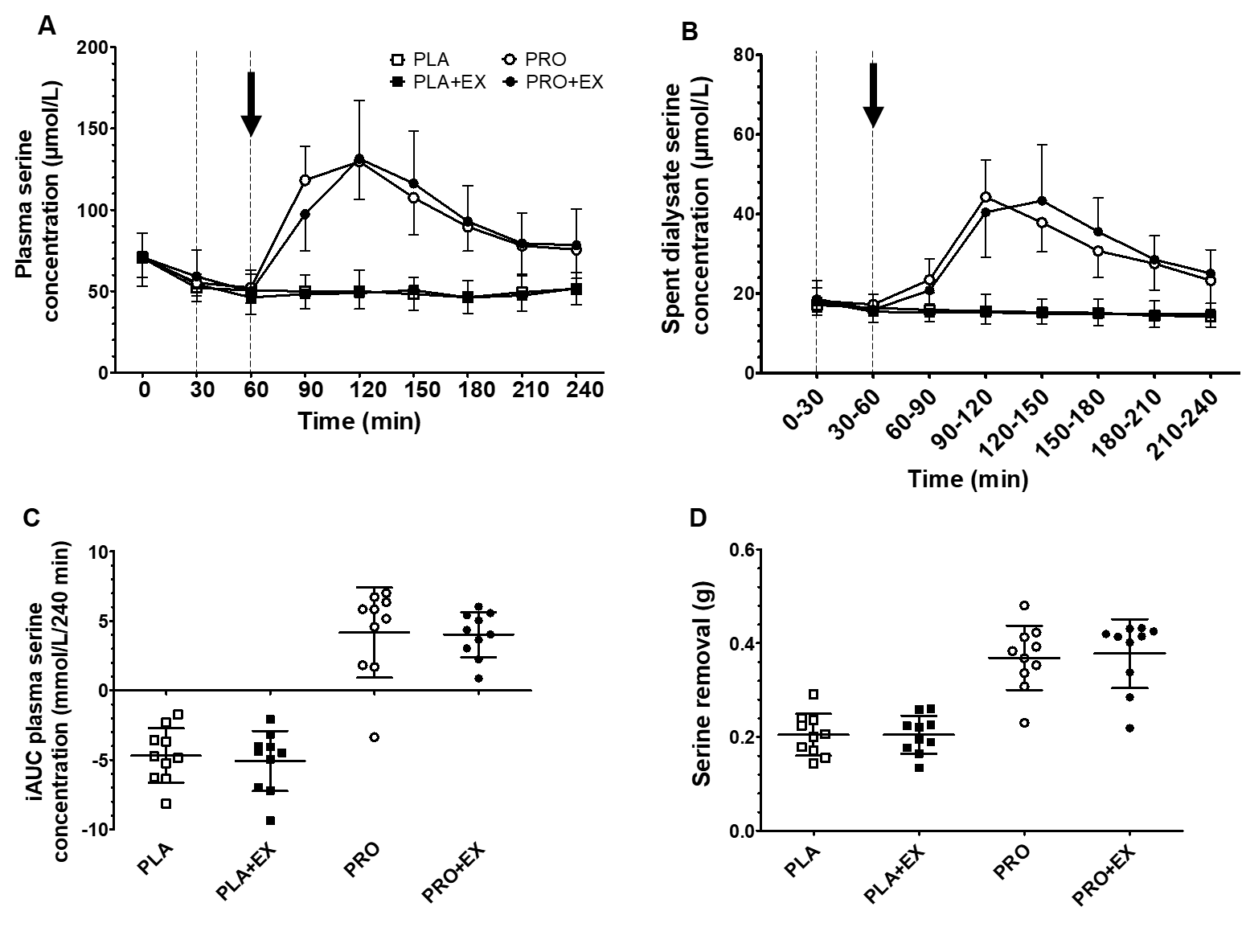


**SUPPLEMENTARY FIGURE 17**

**(A) Plasma serine concentrations, (B) spent dialysate serine concentrations, (C) incremental area under the curve of plasma serine concentrations, and (D) serine removal** **throughout hemodialysis at rest and following exercise with and without protein ingestion**. The dotted lines represent the start and end of intradialytic exercise and the arrow represents the ingestion of the test beverage. Values, *n*=10 for all, are expressed as means±SDs. **A)** Protein *P*<0.001; Exercise *P*=0.848; Time *P*<0.001; Protein × Exercise *P*=0.951; Protein × Time *P*<0.001; Exercise × Time *P*=0.006; Protein × Exercise × Time *P*=0.134. **B)** Protein *P*<0.001; Exercise *P*=0.735; Time *P*<0.001; Protein × Exercise *P*=0.536; Protein × Time *P*<0.001; Exercise × Time *P*=0.049; Protein × Exercise × Time *P*=0.015. **C)** Protein *P*<0.001; Exercise *P*=0.571; Protein × Exercise *P*=0.856. **D)** Protein *P*<0.001; Exercise *P*=0.702; Protein × Exercise *P*=0.595. iAUC, incremental area under the curve; PLA, placebo; PLA+EX, placebo and exercise; PRO, protein; PRO+EX, protein and exercise.


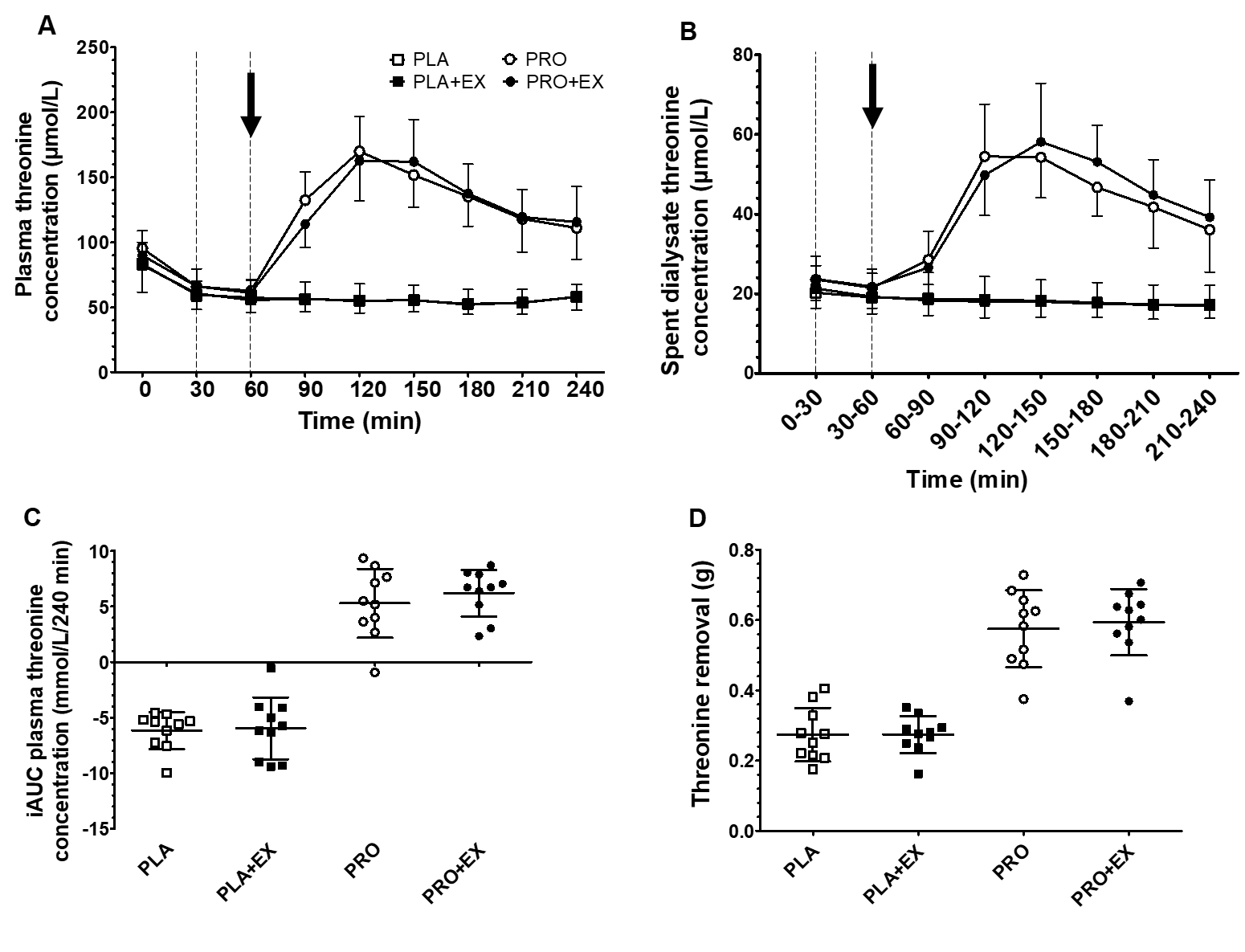


**SUPPLEMENTARY FIGURE 18**

**(A) Plasma threonine concentrations, (B) spent dialysate threonine concentrations, (C) incremental area under the curve of plasma threonine concentrations, and (D) threonine removal** **throughout hemodialysis at rest and following exercise with and without protein ingestion**. The dotted lines represent the start and end of intradialytic exercise and the arrow represents the ingestion of the test beverage. Values, *n*=10 for all, are expressed as means±SDs. **A)** Protein *P*<0.001; Exercise *P*=0.598; Time *P*<0.001; Protein × Exercise *P*=0.619; Protein × Time *P*<0.001; Exercise × Time *P*=0.114; Protein × Exercise × Time *P*=0.140. **B)** Protein *P*<0.001; Exercise *P*=0.568; Time *P*<0.001; Protein × Exercise *P*=0.332; Protein × Time *P*<0.001; Exercise × Time *P*=0.060; Protein × Exercise × Time *P*=0.077. **C)** Protein *P*<0.001; Exercise *P*=0.376; Protein × Exercise *P*=0.660. **D)** Protein *P*<0.001; Exercise *P*=0.538; Protein × Exercise *P*=0.382. iAUC, incremental area under the curve; PLA, placebo; PLA+EX, placebo and exercise; PRO, protein; PRO+EX, protein and exercise.


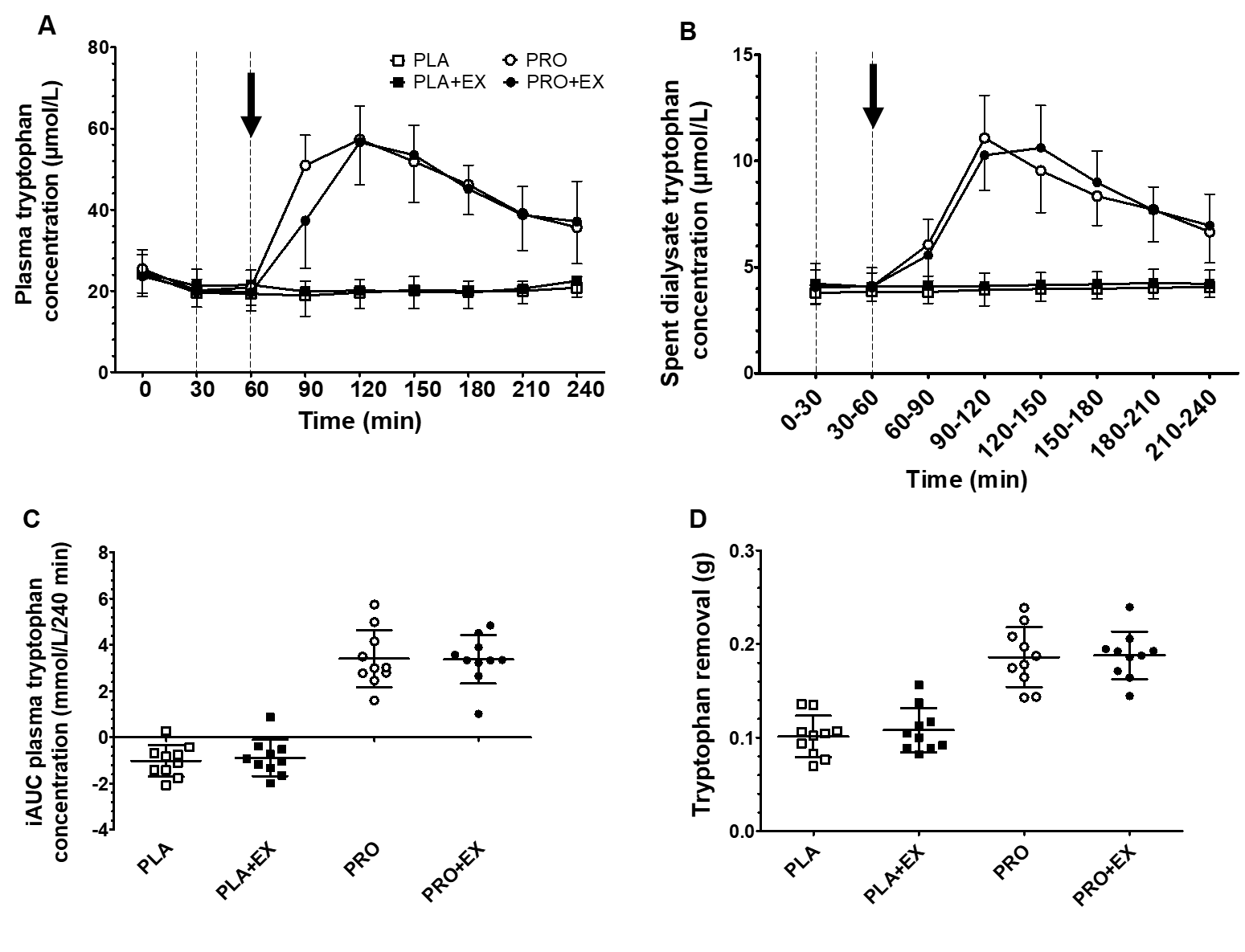


**SUPPLEMENTARY FIGURE 19**

**(A) Plasma tryptophan concentrations, (B) spent dialysate tryptophan concentrations, (C) incremental area under the curve of plasma tryptophan concentrations, and (D) tryptophan removal** **throughout hemodialysis at rest and following exercise with and without protein ingestion**. The dotted lines represent the start and end of intradialytic exercise and the arrow represents the ingestion of the test beverage. Values, *n*=10 for all, are expressed as means±SDs. **A)** Protein *P*<0.001; Exercise *P*=0.476; Time *P*<0.001; Protein × Exercise *P*=0.094; Protein × Time *P*<0.001; Exercise × Time *P*=0.025; Protein × Exercise × Time *P*=0.016. **B)** Protein *P*<0.001; Exercise *P*=0.368; Time *P*<0.001; Protein × Exercise *P*=0.587; Protein × Time *P*<0.001; Exercise × Time *P*=0.061; Protein × Exercise × Time *P*=0.161. **C)** Protein *P*<0.001; Exercise *P*=0.761; Protein × Exercise *P*=0.623. **D)** Protein *P*<0.001; Exercise *P*=0.345; Protein × Exercise *P*=0.548. iAUC, incremental area under the curve; PLA, placebo; PLA+EX, placebo and exercise; PRO, protein; PRO+EX, protein and exercise.


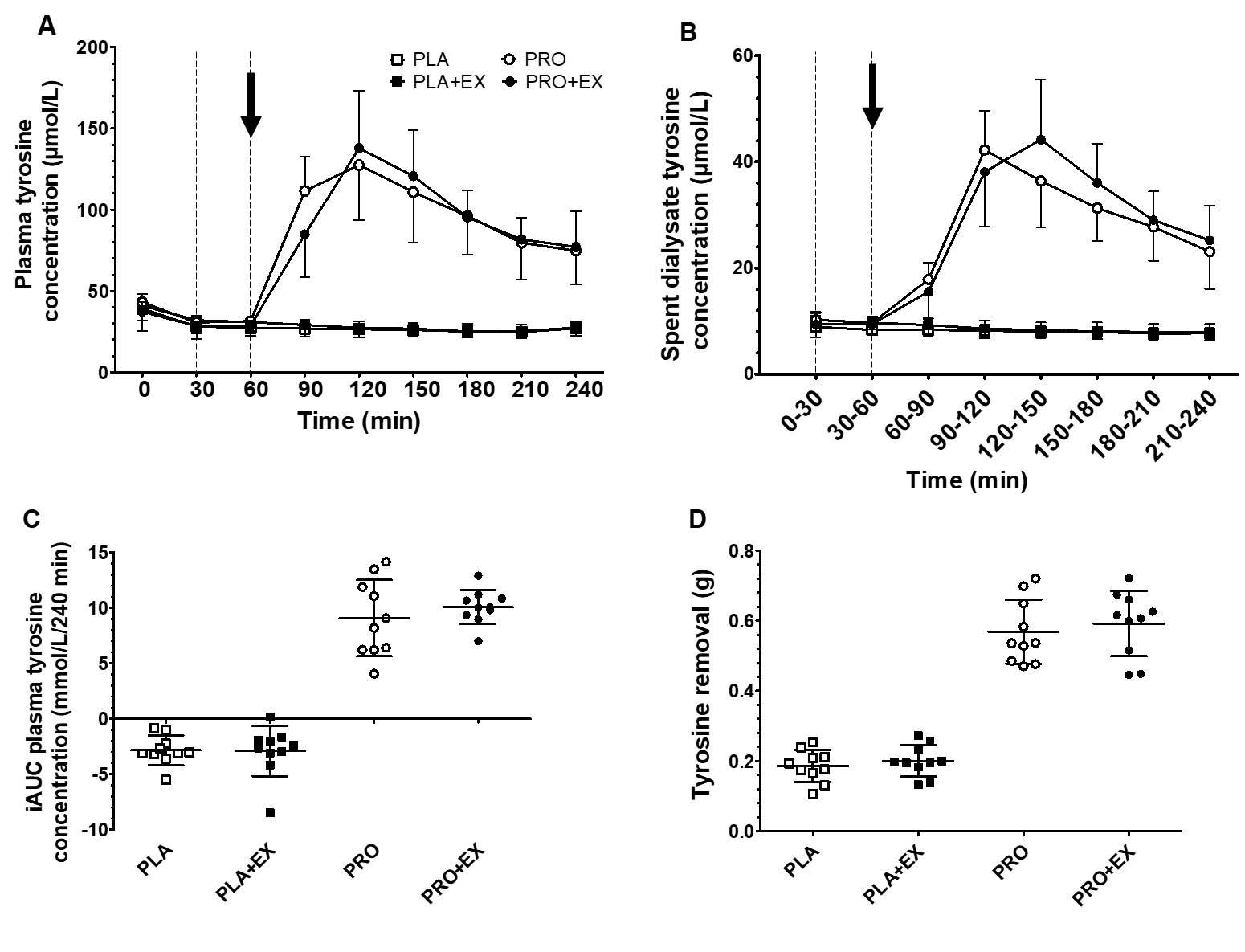


**SUPPLEMENTARY FIGURE 20**

**(A) Plasma tyrosine concentrations, (B) spent dialysate tyrosine concentrations, (C) incremental area under the curve of plasma tyrosine concentrations, and (D) tyrosine removal** **throughout hemodialysis at rest and following exercise with and without protein ingestion**. The dotted lines represent the start and end of intradialytic exercise and the arrow represents the ingestion of the test beverage. Values, *n*=10 for all, are expressed as means±SDs. **A)** Protein *P*<0.001; Exercise *P*=0.987; Time *P*<0.001; Protein × Exercise *P*=0.334; Protein × Time *P*<0.001; Exercise × Time *P*=0.085; Protein × Exercise × Time *P*=0.062. **B)** Protein *P*<0.001; Exercise *P*=0.286; Time *P*<0.001; Protein × Exercise *P*=0.680; Protein × Time *P*<0.001; Exercise × Time *P*=0.069; Protein × Exercise × Time *P*=0.048. **C)** Protein *P*<0.001; Exercise *P*=0.286; Protein × Exercise *P*=0.406 **D)** Protein *P*<0.001; Exercise *P*=0.279; Protein × Exercise *P*=0.730. iAUC, incremental area under the curve; PLA, placebo; PLA+EX, placebo and exercise; PRO, protein; PRO+EX, protein and exercise.

**
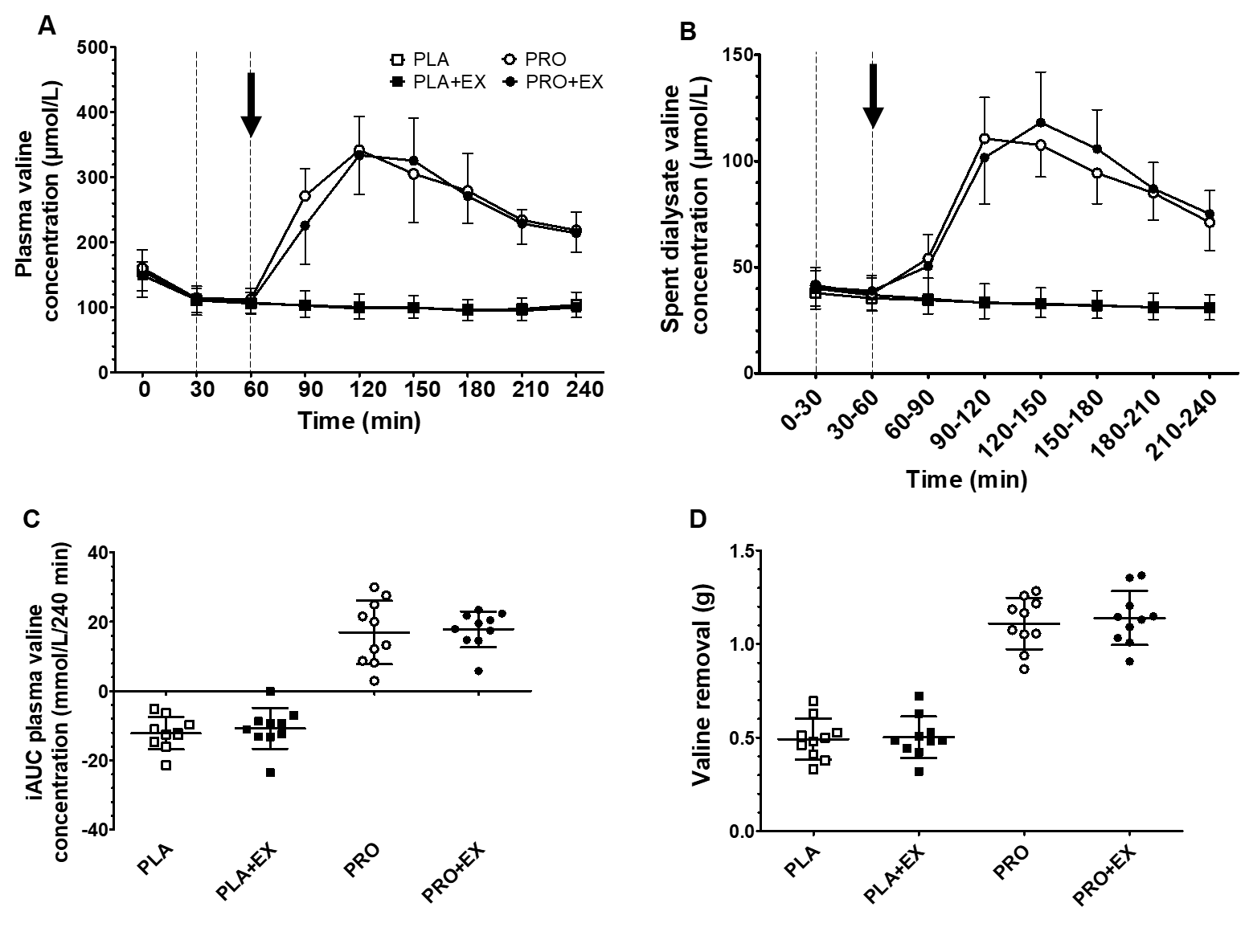
**

**SUPPLEMENTARY FIGURE 21**

**(A) Plasma valine concentrations, (B) spent dialysate valine concentrations, (C) incremental area under the curve of plasma valine concentrations, and (D) valine removal** **throughout hemodialysis at rest and following exercise with and without protein ingestion**. The dotted lines represent the start and end of intradialytic exercise and the arrow represents the ingestion of the test beverage. Values, *n*=10 for all, are expressed as means±SDs. **A)** Protein *P*<0.001; Exercise *P*=0.204; Time *P*<0.001; Protein × Exercise *P*=0.401; Protein × Time *P*<0.001; Exercise × Time *P*=0.099; Protein × Exercise × Time *P*=0.230. **B)** Protein *P*<0.001; Exercise *P*=0.608; Time *P*<0.001; Protein × Exercise *P*=0.674; Protein × Time *P*<0.001; Exercise × Time *P*=0.133; Protein × Exercise × Time *P*=0.117. **C)** Protein *P*<0.001; Exercise *P*=0.461; Protein × Exercise *P*=0.873. **D)** Protein *P*<0.001; Exercise *P*=0.573; Protein × Exercise *P*=0.731. iAUC, incremental area under the curve; PLA, placebo; PLA+EX, placebo and exercise; PRO, protein; PRO+EX, protein and exercise.
